# Wild-Type α-Synuclein Structure and Aggregation: A Comprehensive Coarse-Grained and All-Atom Molecular Dynamics Study

**DOI:** 10.1021/acs.jcim.4c00965

**Published:** 2024-07-24

**Authors:** Gabriel F. Martins, Nuno Galamba

**Affiliations:** † BioISI—Biosystems and Integrative Sciences Institute, Faculty of Sciences of the University of Lisbon, C8, Campo Grande, 1749-016 Lisbon, Portugal

## Abstract

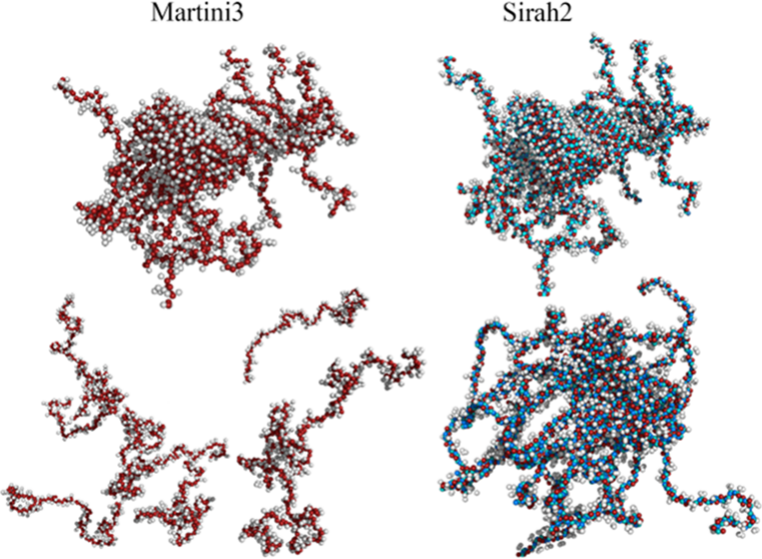

α-Synuclein (α-syn) is a 140 amino acid intrinsically disordered protein (IDP) and the primary component of cytotoxic oligomers implicated in the etiology of Parkinson’s disease (PD). While IDPs lack a stable three-dimensional structure, they sample a heterogeneous ensemble of conformations that can, in principle, be assessed through molecular dynamics simulations. However, describing the structure and aggregation of large IDPs is challenging due to force field (FF) accuracy and sampling limitations. To cope with the latter, coarse-grained (CG) FFs emerge as a potential alternative at the expense of atomic detail loss. Whereas CG models can accurately describe the structure of the monomer, less is known about aggregation. The latter is key for assessing aggregation pathways and designing aggregation inhibitor drugs. Herein, we investigate the structure and dynamics of α-syn using different resolution CG (Martini3 and Sirah2) and all-atom (Amber99sb and Charmm36m) FFs to gain insight into the differences and resemblances between these models. The dependence of the magnitude of protein–water interactions and the putative need for enhanced sampling (replica exchange) methods in CG simulations are analyzed to distinguish between force field accuracy and sampling limitations. The stability of the CG models of an α-syn fibril was also investigated. Additionally, α-syn aggregation was studied through umbrella sampling for the CG models and CG/all-atom models for an 11-mer peptide (NACore) from an amyloidogenic domain of α-syn. Our results show that despite the α-syn structures of Martini3 and Sirah2 with enhanced protein–water interactions being similar, major differences exist concerning aggregation. The Martini3 fibril is not stable, and the binding free energy of α-syn and NACore is positive, opposite to Sirah2. Sirah2 peptides in a zwitterionic form, in turn, display termini interactions that are too strong, resulting in end-to-end orientation. Sirah2, with enhanced protein–water interactions and neutral termini, provides, however, a peptide aggregation free energy profile similar to that found with all-atom models. Overall, we find that Sirah2 with enhanced protein–water interactions is suitable for studying protein–protein and protein–drug aggregation.

## 1 Introduction

Protein aggregation has been implicated in several neurodegenerative diseases such as Alzheimer’s disease (AD) and Parkinson’s disease (PD).^1−4^ PD and other synucleinopathies, in particular, have been linked with the formation of cytotoxic oligomers,^5−8^ primarily composed of α-synuclein (α-syn), intermixed with membranous organelles^9^ that accumulate in neuronal intracytoplasmic inclusions, called Lewy bodies and Lewy neurites.^10,11^ Although the cytotoxicity mechanism remains elusive, these abnormal aggregates are thought to be the main culprit for the loss of dopaminergic neurons in the substantia nigra pars compacta.^12,13^

**Figure 1:**
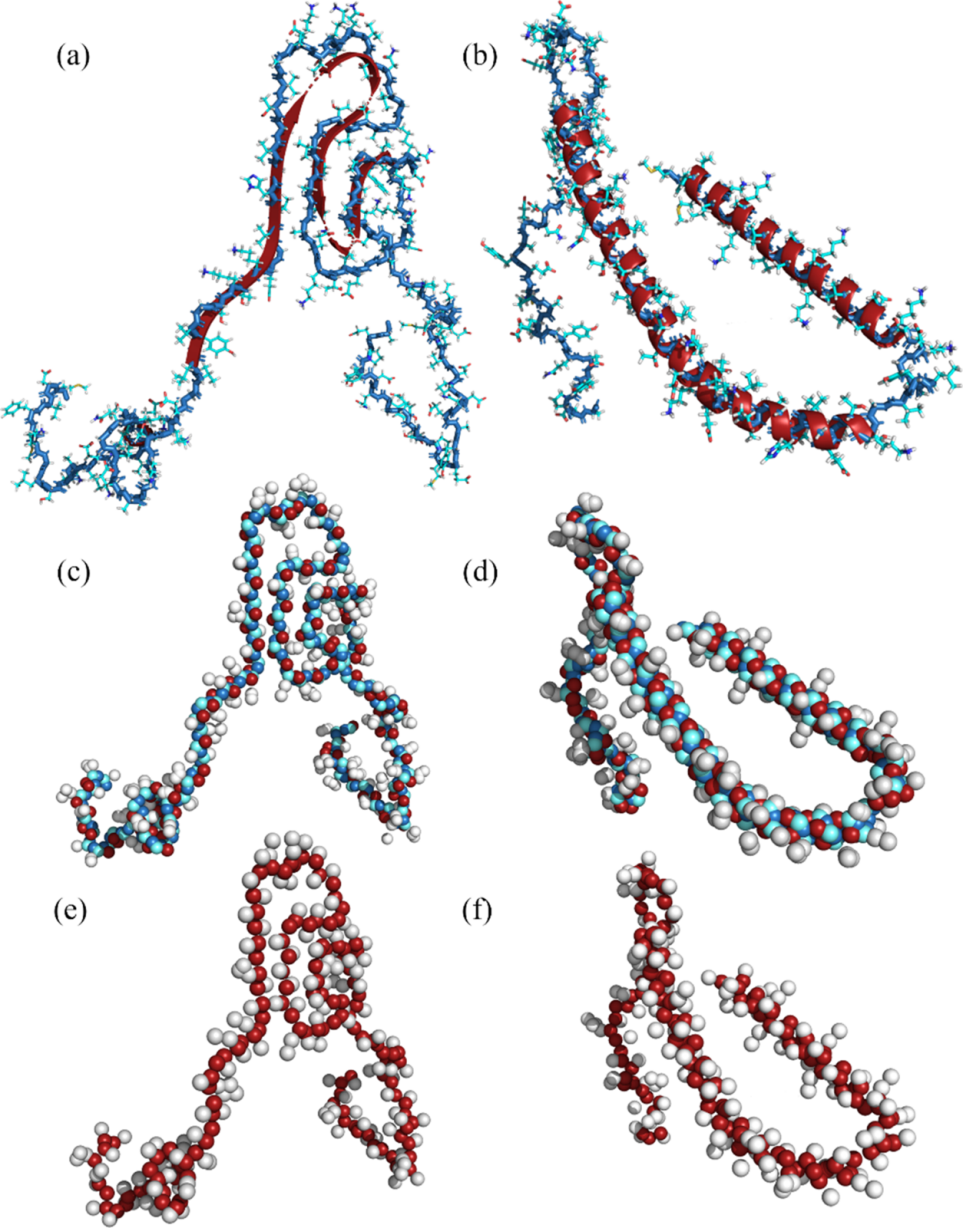
Molecular representation of α-syn using an all-atom model for the monomer in the (a) fibril (2n0a) and (b) bound to a micelle (2kkw); Sirah2 in the (c) fibril and (d) bound to a micelle; and Martini3 in the (e) fibril and (f) bound to a micelle. These structures were used to probe the dependence of the starting conformation for all of the FFs investigated. Red and light and dark blue beads represent the backbone in Sirah2; red beads represent the backbone in Martini3.

α-Syn is a 140 amino acid intrinsically disordered protein (IDP) mainly expressed in the central nervous system.^14−16^ The protein can be separated into three distinct domains: the N-terminal (N-term), a membrane-binding domain that tends to form α-helices,^17^ encompassing amino acids 1–60, a hydrophobic and amyloidogenic sequence involving amino acids 61–95, the so-called nonamyloid-β component (NAC),^18^ and the C-terminal (C-term) domain, a more disordered region composed of the amino acids 96–140.^14^ While IDPs lack a well-defined three-dimensional conformation, they sample a complex spatiotemporal heterogeneous ensemble of conformations. For instance, when bound to a membrane, α-syn adopts partially folded structures (see Figure 1), whereas in solution, more disordered conformations are observed.^17,19,20^ While these multiple conformations are difficult to assess experimentally, molecular dynamics (MD) simulations provide an alternative route to investigate the conformational space of IDPs as well as their aggregation pathways. Understanding the relationship between the monomer conformational transformations underlying nucleation and subsequent oligomerization and how these relate to the disease is pivotal to the development of aggregation inhibitors.^21^ Thus, various all-atom^22−40^ and coarse-grained (CG)^37,41−50^ molecular simulations studies were reported on the structure of the monomer and small oligomers of α-syn. Table S1 provides details on the models and systems studied in these works. The accuracy of molecular simulations depends on the accuracy of the force fields (FFs) used to describe intra- and intermolecular interactions in addition to statistical sampling. However, because typical protein FFs were primarily developed to model globular proteins, it is not unexpected that they cannot reproduce the conformational space of IDPs, in general, and various modifications were carried out to address the disordered nature of these proteins.^25,51−56^ These include, for instance, the modification of torsion potentials^54,57^ and the redefinition of protein–water interactions.^25,55^ The size and elongated structure of some proteins, such as α-syn, represent an additional difficulty, as large simulation boxes are required to avoid spurious interactions with image proteins under a periodic boundary conditions framework. Thus, CG FFs emerge as promising alternatives, allowing simulating larger proteins for longer periods of time at the cost of the loss of atomic detail. For a recent review on CG models, see ref (58). Since CG models do not account for high frequency bonds, the timesteps associated with these calculations are higher by a decade than those in atomistic descriptions. Hence, some CG FFs were also modified to study IDPs, and α-syn in particular.^44,46,48,59^ Nevertheless, although these can provide a good description of the monomer’s structure, the applicability of these FFs to study protein–protein aggregation or protein–drug interactions has been much less explored.

Here, we study the structure and dynamics of the α-syn monomer using two CG models, Martini^60−62^ and Sirah,^63,64^ and two all-atom FFs, Amber99sb^65^ and Charmm36m^54^ with different water models (TIP3P,^66^ TIP3SP,^54^ TIP4P-Ew,^67^ OPC^68^). The influence of protein–water interactions was assessed in CG simulations by using previously proposed reparameterizations.^46,48^ For Sirah2, an increase of 30% on those interactions allowed reproducing the experimental radius of gyration, chemical shifts, secondary structures, and long-range contacts of the monomer of α-syn.^46^ For Martini3, in turn, a 10% increase on protein–water interactions reproduced small-angle X-ray scattering and paramagnetic relaxation enhancement data.^48^ The specific choice of these CG models was related to the good agreement with experimental data found in these works for the monomer of α-syn.

The dimerization of CG α-syn was studied here through umbrella sampling along with the stability of a protofiber comprised of 10 α-syn proteins. Further, the binding free energy of NACore,^69^ an 11-mer peptide from the NAC domain, was studied through umbrella sampling, with both CG and all-atom FFs. Additionally, we assessed the need to use enhanced sampling methods, in particular, Hamiltonian replica exchange, to sample the conformational space of a CG model of α-syn. This allows understanding whether enhanced protein–water (or weakened protein–protein) interactions are fundamental to observe structures consistent with those found through experiments or limitations are, instead, mostly related to sampling.

## 2 Methods and Theory

### 2.1 Force Fields

The α-syn monomer was studied using the CG models, Martini3 (M3)^60−62^ and Sirah2 (S2),^63,64^ and the all-atom models, Amber99sb^65^ and Charmm36m.^54^ These CG FFs (FFs) were chosen because of their different molecular resolutions and the fact that both have been previously used to study α-syn. The original parametrizations and scaled versions,^46,48^ shown to provide an improved description of the monomer of α-syn, were investigated. The latter are hereafter represented by Martini3* (M3*) and Sirah2* (S2*), respectively. For M3*, protein–water interactions are scaled by a factor of 1.1,^48^ whereas for S2*, protein–water interactions are scaled by a factor of 1.3.^46^ We note that in ref (46), the simulations were performed with Sirah1;^63^ however, we found similar results using this scaling for the newest version, Sirah2. The enhancement of protein–water interactions results in less compact structures, characterized by a radius of gyration closer to the available experimental^70−74^ values (2.5–4.0 nm).

For Amber99sb, two water models were investigated, namely, TIP4P-Ew^67^ and OPC.^68^ For Charmm36m, the original TIP3P^66^ and TIPS3P^54^ were investigated. The latter includes van der Waals parameters for the H atom, opposite to the original TIP3P water model. The Lennard-Jones^12-6^ (LJ^12-6^) parameters in TIPS3P are, however, σ_H_ = 0.04 nm and ε_H_ is 0.4184 kJ mol^–1^, instead of σ_H_ = 0.04 nm and ε_H_ = 0.1925 kJ mol^–1^ in the original CHARMM modified model mTIP3P.

Figure 1 shows conformations of the α-syn monomer in a fibril (2n0a)^75^ and bound to a micelle of sodium lauroyl sarcosinate (2kkw)^76^ at three different representation levels, all-atom, S2, and M3. The 2kkw structure is a partially folded conformation, characteristic of a membrane-bonded α-syn, in which the N-terminal domain adopts an α-helical conformation upon membrane binding.

M3 is a low-resolution model, whereas S2 has a hybrid resolution between a low-resolution CG and an all-atom description. S2 uses a standard pairwise Hamiltonian that represents each backbone amino acid with three beads in the position of nitrogen, carbonyl, and C_α_ atoms. The side chains are represented with a lower resolution with one-to-five beads depending on the residues’ side chain. These beads have a pseudocharge that changes according to the number of hydrogen bond acceptors of each residue and helps stabilize secondary structures through hydrogen bond-like interactions. The dihedral angles also play a role in the definition of the secondary structure of the system, forcing the existence of both α-helices and β-strands conformations.^63^ S2 is a structurally unbiased model, optimized to be used with an explicit model of water, the WT4,^77^ in which four interconnected beads, with tetrahedral geometry and a partial charge, account for approximately 11 water molecules. The main purpose is to achieve faster simulations than atomistic calculations while maintaining traits that are lost to other CG FFs, such as the use of implicit solvents that use uniform dielectric constants, the biased imposed secondary structures, or the lack of long-range electrostatic interactions.

The mapping in Martini is based on a four-to-one approach (i.e., four heavy atoms are represented by a single interaction center). In the case of amino acids, the representation can go up to a five-bead representation, and the backbone is represented by one bead placed at the center of mass (COM) of the amino acid backbone. Ringlike molecules, however, are mapped with higher resolution (up to two-to-one).^61^ The M3 FF covers new bead types and readjusts several nonbonded interactions when compared to the Martini2 version, leading to an improved accuracy of other types of biological systems.^62^ The standard water in Martini is represented by a single bead, a neutral particle that interacts with other particles in the system by LJ^12-6^ parameters, although a polarizable water model (not explored here) has also been proposed.^78^ The standard water can have different sizes, namely, regular (RW), small (SW), or tiny (TW). Here, the standard RW water model was used.

### 2.2 Molecular Dynamics

Molecular dynamics (MD) simulations of α-syn in a 0.1 M NaCl aqueous solution were carried out in the isothermal–isobaric (*N*,*p*,*T*) ensemble at 310 K and 0.1 MPa with the GROMACS program.^79^ The temperature (*T*) and pressure (*p*) were controlled with the Nosé–Hoover thermostat^80,81^ and the Parrinello–Rahman barostat,^82^ and the equations of motion were solved using the Verlet leapfrog algorithm with 20 and 2 fs timesteps for the CG and all-atom models, respectively. The α-syn was simulated in a cubic box with periodic boundary conditions. Long-range corrections were applied to the pressure and the potential energy (PE). For M3, electrostatic interactions were computed with a reaction field (RF); preliminary simulations with both RF and particle-mesh Ewald^83^ (PME) methods showed similar results for the root-mean-square deviation (RMSD) and the radius of gyration (*R*_g_) of α-syn. A dielectric constant of 15 and a cutoff of 1.1 nm was used for the nonbonded van der Waals and electrostatic interactions. These same values were used in the development of Martini3^62^ and in the work by Thomasen et al. for α-syn.^48^

For Sirah2, electrostatic interactions were computed via the PME method. A 1.2 nm cutoff was used for the nonbonded van der Waals interactions and the PME real space electrostatic interactions. A similar cutoff was used in the development of Sirah2^64^ and in the work by Ramis et al. for α-syn.^46^ A similar approach was followed for the all-atom MD simulations. The electrostatic interactions were computed with the PME method, and a 1.0 nm cutoff was used for the nonbonded van der Waals interactions and the PME real space electrostatic interactions.

Following the steepest descent energy minimization, the solvent was equilibrated for 10 ns in the *NVT* ensemble applying harmonic restraints to the CG protein; 1000 kJ mol^–1^ nm^–2^ harmonic force constants were used to restrain the beads. A short 5 ns equilibrium in the *NpT* ensemble was carried out by also applying harmonic restraints to the protein, and the trajectories were then propagated in the *NpT* ensemble without restraints for another 10 μs in water. For all-atom MD simulations, the systems were equilibrated for 250 ps in the *NVT* ensemble and 500 ps in the *NPT* ensemble, with harmonic restraints in the protein. The trajectory was then propagated in the *NpT* ensemble for 1 μs for Amber99sb and 500 ns for Charmm36m.

MD simulations of a fibril composed of 10 monomers of α-syn (2n0a)^75^ were also performed with S2* and M3* using similar conditions as for the monomers, although a longer equilibration of 50 ns was carried out in the *NpT* ensemble with similar harmonic restraints applied to the fibril. The *NpT* production trajectories were then propagated for 4 μs.

### 2.3 Replica Exchange Solute Tempering 2

CG FFs have a smoother potential energy surface compared with all-atom FFs. Nonetheless, some previous works,^42,46^ including using Sirah,^46^ used enhanced sampling techniques to study α-syn’s conformational space modeled by CG models. To assess the potential need for enhanced sampling methods, replica exchange (aka parallel tempering) solute tempering 2 (REST2)^84,85^ was used to simulate S2 and S2* models of α-syn. This allows an understanding of the extent to which conformations found with S2*, through ordinary MD, are observed with S2 using REST2.

For the all-atom models, REST2 was not used because of the computational cost, whereas for Martini, the reduced number of charged beads and the absence of backbone dihedrals preclude this method from efficiently sampling different replicas. For this reason, the potential energy surface is less rough than those for all-atom models and Sirah, and ordinary MD simulation should allow ergodic sampling.

The replica exchange method (REM) allows a system to escape local energy minima by exchanging conformations between adjacent replicas of the same system simulated at different temperatures^86,87^ and/or Hamiltonians^84,85,88^ using the Metropolis Monte Carlo algorithm. REST2^85^ is a Hamiltonian replica exchange method (H-REM) as opposed to the temperature-replica exchange method (T-REM). Thus, in REST2, all of the replicas are simulated at a single temperature (i.e., the temperature of interest, *T*_0_ = 310 K), but except for the lowest replica (i.e., replica 0), the other replicas are simulated on different potential energy surfaces (without physical meaning).

The probability of configuration *X* in the *m*th replica at *T*_*m*_ is given by
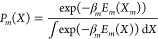
1where *E*_*m*_ is the potential energy of replica *m*, β = 1/*k*_B_*T*, *k*_B_ is the Boltzmann constant, and the denominator of eq 1 is the respective configurational partition function. The joint probability distribution for the *M* replicas is given by the product of the probability of each replica^88^
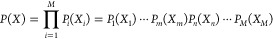
2

and the detailed balance condition for the transition between replicas *m* and *n* is given by^85,89^
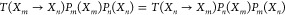
3where *T*(*X*_*m*_ → *X*_*n*_) and *T*(*X*_*n*_ → *X*_*m*_) are the transition probabilities for the exchange of configurations *X*_*m*_ and *X*_*n*_ between replicas *m* and *n*. The ratio of transition probabilities is^85,89^
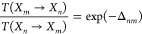
4

with
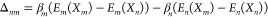
5

In REST2 (as in REST1^90^), the potential energy is split into three contributions, namely, protein intramolecular potential energy, *E*_pp_(*X*), protein–water, *E*_pw_(*X*), and water–water potential energy, *E*_ww_(*X*). These are scaled as
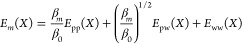
6

From eq 6 and Metropolis Monte Carlo algorithm, the following equation can be derived for the acceptance probability^85^
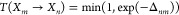
7

with
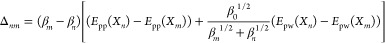
8that is, the transition acceptance probability is equal to 1 for Δ_nm_ ≤ 0 and exp(−Δ_nm_) for Δ_nm_ > 0. Notice that the water–water potential energy, *E*_ww_(*X*), is no longer part of the acceptance probability, opposite to T-REM (see eq 5). This is one of the reasons for the increased rate of acceptance and, therefore, the need for a lower number of replicas in REST2 relative to T-REM. For a detailed discussion about the limitations of REST1, we refer to the original article of REST2.^85^

In REST2, the *E*_pp_ bonded interaction energy terms (proper dihedrals) are scaled by (β_*m*_/β_0_), the *E*_pp_ Lennard-Jones ε parameters are scaled by (β_*m*_/β_0_), the *E*_pp_ charges are scaled by (β_*m*_/β_0_)^1/2^, and the *E*_pw_ cross terms are scaled by (β_*m*_/β_0_)^1/2^. Although, unlike REST1, only the potential energy is different among the different replicas, whereas the temperature remains the same, “hot” and “cold” regions^84^ must be defined in terms of the potential energy scaling.^85^ Here, the hot region was chosen to be the protein and the ions, whereas the cold region was water. A similar approach was also followed by Ramis et al.^46^ Notice that α-syn has a charge of −9*e*; hence, scaling of the charges of the protein alone would result in charged replicas of different charges. Although a neutralizing background can be added in PME,^84^ this would result in different hot–cold interactions. Thus, eq 6 is rewritten in the form
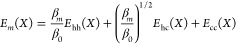
9where ion–ion interactions are included in the hot region, and, therefore, *E*_hh_ = *E*_pp_ + *E*_ii_ + *E*_pi_ and *E*_hc_ = *E*_pw_ + *E*_iw_; here, h, c, and i stand, respectively, for hot, cold, and ion. Thus, interactions inside the hot region are kept at an effective temperature (β_*m*_/β_0_), those between the hot and the cold regions are kept at an effective intermediate temperature (β_*m*_/β_0_)^1/2^, and interactions in the cold region are kept at temperature β_0_.^84^ REST2 MD were carried out for S2 and S2* using 30 replicas with effective temperatures ranging between 310 and 450 (see Table S2). This choice was based on a previous work on REST2 for Sirah2^46^ to allow a direct comparison with those results. Exchanges were attempted every 2 ps with an average acceptance ratio of about 15–20%.

Since Sirah2 is a “hybrid-resolution” model that keeps a resemblance with all-atom models, the scaling process accounted for both protein dihedral angles and nonbonded van der Waals and electrostatic bead interactions. The REST2 simulations were performed with GROMACS patched with PLUMED,^91^ as implemented by Bussi^92^ under the same temperature and pressure conditions of ordinary MD simulation.

### 2.4 Umbrella Sampling

We calculated the potential of mean force^93^ (PMF), aka the aggregation free energy profile, of α-syn for both S2* and M3* and an 11-mer segment, coined NACore^69^ (_68_GAVVTGVTAVA_78_), relevant to the aggregation and cytotoxicity of α-syn, for S2, S2*, M3, M3*, as well as Aamber99sb and Charmm36m. The PMFs were computed through umbrella sampling.^94,95^ The reaction coordinate, ξ, was chosen to be the distance between the center of mass (COM) of the “central” amino acid in α-syn and that in NACore. The PMF is given (up to a constant *C*) by^96−98^
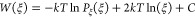
10where *P*_ξ_ is the probability distribution along the reaction coordinate ξ.
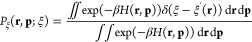
11

The second term in eq 10 accounts for the transformation between Cartesian coordinates to internal coordinates (i.e., the amino acid–amino acid COM distance). This is obtained from the respective Jacobian and accounts for the increasing sampling volume with ξ in the spherical polar coordinates. This term is therefore an entropic correction to the free energy along ξ.

In umbrella sampling, a biased (b) probability distribution along ξ is calculated by adding a bias potential to the Hamiltonian, restraining the sampling along ξ
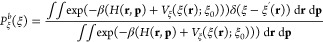
12

A harmonic potential with a spring constant, *K*_ξ_, of 1000 kJ mol^–1^ nm^–2^ was used to restrain ξ
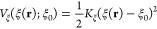
13and a spacing of 0.05 nm between windows (umbrellas) was used. The PMF for a window *i* is given by
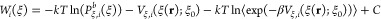
14

The third term on the right-hand side (ensemble average), commonly represented by *F*_*i*_, is the free energy shift in umbrella *i* due to the bias potential. This was estimated through the weighted histogram analysis method^99^ (WHAM) to obtain the unbiased PMF. The PMFs were then shifted to have zero free energy at large separation distances (i.e.,  was used to define *C*), and the Bayesian bootstrap method^100^ was used to estimate the PMFs errors.

The umbrella sampling MD simulations for NACore with Martini3 (M3 and M3*) and Sirah2 (S2 and S2*) were performed for 1 μs in the *NpT* ensemble after steepest descent energy minimization, a 100 ps equilibration in the *NVT* ensemble, and a 100 ns equilibration in the *NpT* ensemble. These simulations amounted to 63 μs, corresponding to 63 windows. The umbrella sampling MD simulations for NACore with the all-atom FFs were performed for 70 ns in the *NpT* ensemble after the steepest descent energy minimization, a 100 ps equilibration in the *NVT* ensemble, and a 20 ns equilibration in the *NpT* ensemble. These amounted to over 4.4 μs, corresponding to 63 windows.

The MD simulations for α-syn with M3 and M3*, and S2 and S2*, were performed for 700 ns in the *NpT* ensemble after steepest descent energy minimization, a 100 ps equilibration in the *NVT* ensemble, and 100 ns equilibration in the *NpT* ensemble. These amounted to 63 μs, corresponding to 90 windows for M3 and M3* and 70 μs for S2 and S2*, corresponding to 100 windows.

The umbrella sampling starting configurations of the peptide and α-syn at each ξ value were obtained through steered MD simulation with a spring constant of 1000 kJ mol^–1^ nm^–2^ and a pull rate of 0.01 nm ps^–1^.

### 2.5 Principal Component Analysis

Principal component analysis (PCA) is a widely used linear dimensionality reduction method that systematically transforms coordinates within each trajectory’s frame into a linear combination of orthogonal vectors, known as principal components. The initial vectors/components capture the utmost variance in the original data, which translates to the most uncorrelated variance in the analyzed trajectory. Subsequent components successively contribute to diminishing amounts of variance, creating a hierarchical representation of the underlying data structure.^101^ For IDPs, large variances are expected, resulting in many important eigenvectors or principal components.

Here, the nonmass weighted covariance matrix, *C*, for the C_α_ atomic displacements of the all-atom and the CG backbone beads of α-syn is a 420 × 420 matrix encompassing displacements along the three Cartesian components for the 140 C_α_ atoms or backbone beads. The matrix C is given by^101^
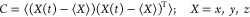
15where *X* are the instantaneous coordinates and ⟨*X*⟩ their respective averages, (*X*(*t*) – ⟨*X*⟩) and (*X*(*t*) – ⟨*X*⟩)^T^ are, respectively, column and row (transpose) matrices, and the elements of the covariance matrix are
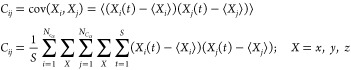
16where *N*_c_α__ is the number of C_α_ or beads and *S* is the number of MD simulation configurations analyzed. The elements of *C* were calculated after a least-squares fit of the protein coordinates with respect to the crystal structure to remove the protein’s roto-translation motions in the MD simulation box. The matrix *C* is symmetric and can be diagonalized through an orthogonal transformation^101^
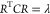
17resulting in 440 eigenvectors and eigenvalues, where the eigenvectors are given by the columns of *R*, *R*^T^ is the transpose matrix, and λ is a diagonal matrix with the respective eigenvalues ordered by descending order.

The subspace formed by zero or close to zero eigenvalues (i.e., mean square displacements) corresponds essentially to the protein’s forbidden motion subspace.^101^ That formed by the remaining small eigenvectors represents low amplitude fluctuations already outside the “essential” subspace. Thus, the trajectory can be analyzed regarding a few principal components (i.e., eigenvectors) where differences in the first component are more important than in the second and so on. Here, analysis was limited to the first two eigenvectors for simplicity, although, as discussed in the Section 3, these encompass a relatively small fraction of the protein conformational space, as expected.

## 3 Results and Discussion

### 3.1 Structure of α-Syn

**Figure 2:**
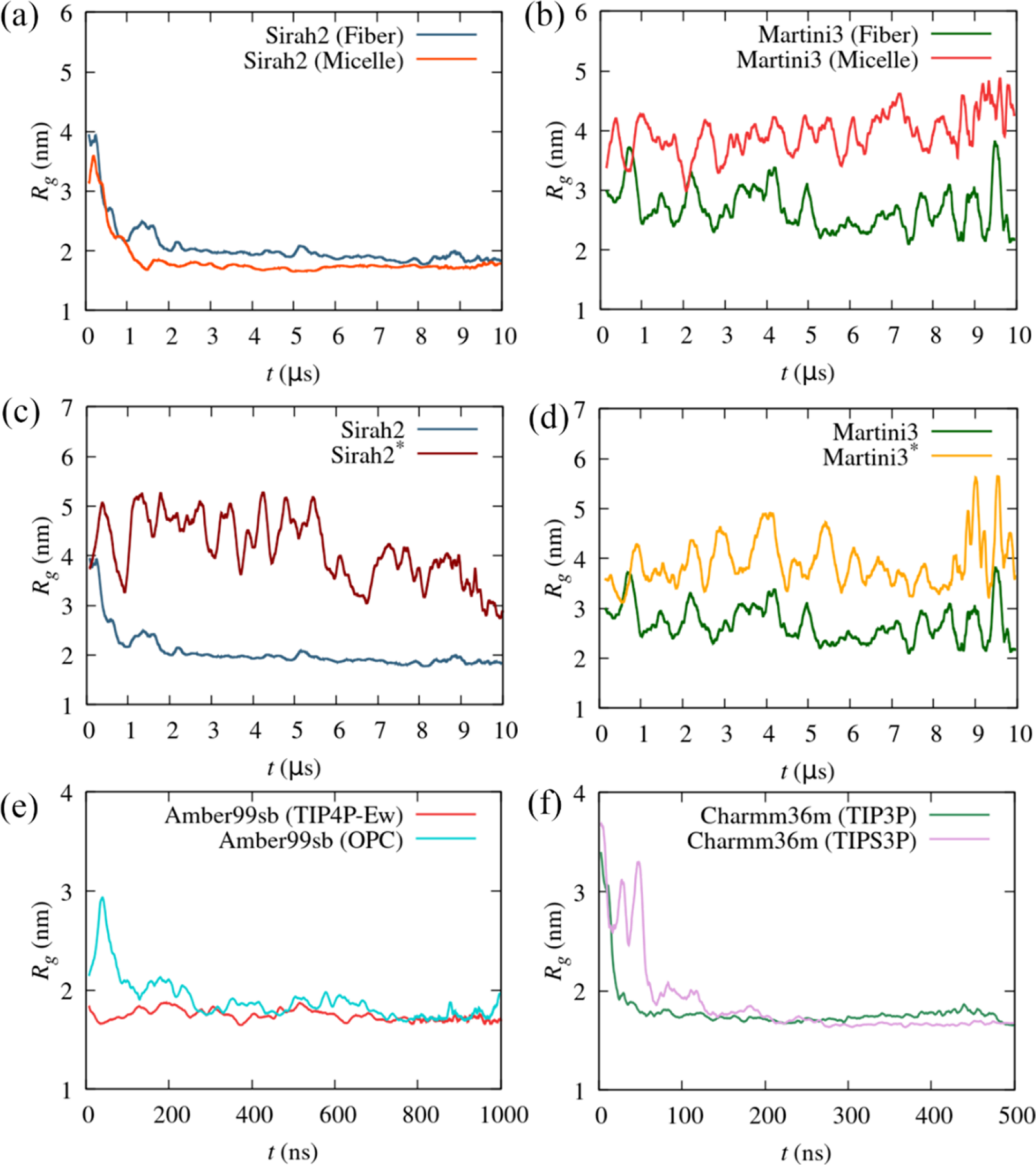
Moving average (MA) of the radius of gyration (*R*_g_) of α-syn as a function of time for (a) Sirah2 and (b) Martini3, starting from a monomer in the fibril (2n0a) and a monomer bound to a micelle (2kkw); the different order between the *R*_g_ for the fiber and micelle structures at short times in passing from Sirah2 (a) to Martini3 (b) results only from the MA calculation; (c) MA of the *R*_g_ for Sirah2 and Sirah2* starting from a monomer in the fibril; (d) MA of the *R*_g_ for Martini3 and Martini3* starting from a monomer in the fibril; (e) MA of the *R*_g_ for Amber99sb with TIP4P-Ew and OPC starting from a monomer in the fibril*;* and (f) MA of the *R*_g_ for Charmm36m with TIP3P and modified TIPS3P starting from a monomer in the fibril.

We start by discussing the possible dependence of the starting conformation (Figure 1) through unbiased (i.e., ordinary) MD simulation of the CG models of α-syn. Figure 2a suggests ergodic sampling for S2 within 10 μs, as opposed to M3 (Figure 2b), for which a dependence of the initial conformation is observed. We performed three replicates and found similar results (see Figure S1). This seemingly quasi-ergodic sampling of M3 is unexpected since the potential energy surface should be significantly smoother than for S2. Although longer trajectories could result in convergence of the average *R*_g_ to a similar range of values, this was not pursued here.

The influence of protein–water interactions on the structure of the CG models of α-syn can be seen in Figure 2c,d, which shows an increase of the *R*_g_ for the scaled versions S2* and M3*, as expected, since protein–water interactions are favored in the latter. More importantly, larger fluctuations are observed, reflecting the transformation between compact and extended conformations not observed in S2. Experimental values of the *R*_g_ vary between 2.5 and 4.0 nm.^70−74^ These results are consistent with previous studies reported in the literature for M3^48^ and Sirah1,^46^ where the respective protein–water scaling factors were proposed.

Figure 2e,f shows good concordance among the different all-atom FFs. Values of *R*_g_ similar to those for S2 are observed even for Charmm36m. Fully atomistic FFs generally result in exceeding stabilization of compact conformations associated with globular proteins, reflected in lower *R*_g_, compared to experimental values. Thus, all-atom FFs and S2 predict more compact structures and the absence of any significant conformational transformations. However, scaling of S2 already provides values within the experimental interval and larger fluctuations. For the all-atom FFs, although protein–water interactions were varied to some extent, by using different water models, no significant differences were observed. This is not unexpected for Amber99sb, since previous all-atom simulations showed that modifications had to be carried out in the protein and water models to reproduce experimental NMR and *R*_g_ data.^25,29,57^ More surprising are the results for Charmm36m since this FF was already modified to reproduce the structure of folded and disordered proteins. We stress, nonetheless, that the results for the all-atom FFs are also associated with sampling limitations. However, the fact that α-syn can adopt extended conformations at high effective temperatures (or temperatures) requires the use of very large MD simulation boxes, deeming the method very demanding from a computational viewpoint for all-atom MD simulations.

Figures S2 and S3 provide similar plots for the root-mean-square deviation (RMSD) and the root-mean-square fluctuations (RMSF). The S2 and M3 RMSD show a slight dependence on the starting conformation. On the other hand, nearly no differences are observed between the RMSD for S2 and S2*, and between M3 and M3*, although larger fluctuations are observed for S2* and M3*, as expected. Similar RMSD are also observed among the different all-atom FFs.

The RMSF of M3 shows a clear dependence of the starting conformation difference, consistent with the *R*_g_. These differences are especially marked in the first amino acids of the N-terminal region, with especially large values for the micelle-bond starting conformation. This indicates that this domain is largely responsible for the increased *R*_g_ values depicted in Figure 2. Protein–water interaction enhancement in S2* and M3* leads to an increase of the RMSF, as expected. For the all-atom FFs, more similar values are found, with slightly larger values for some amino acid sequences in Amber99sb/OPC and Charmm36m/TIP3P.

### 3.2 Replica Exchange

**Figure 3:**
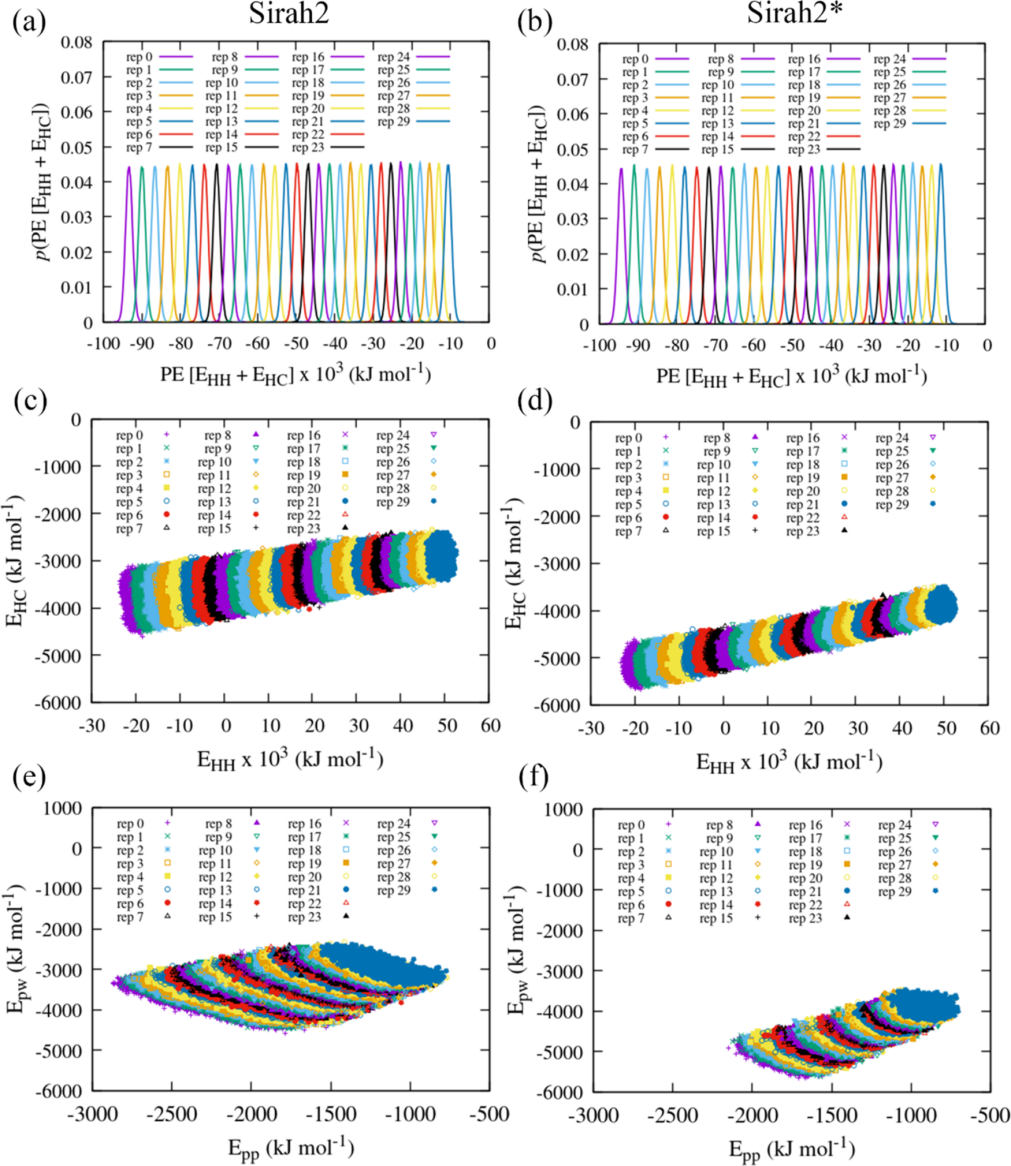
REST2 hot–hot (HH) and hot–cold (HC) potential energy (PE) distributions for the different replicas for (a) Sirah2 and (b) Sirah2*; HC PE as a function of the HH PE for (c) Sirah2 and (d) Sirah2*; protein–water PE as a function of the protein–protein PE for (e) Sirah2 and (f) Sirah2*.

To evaluate the potential need for using enhanced sampling methods for CG models, we also studied the structure of S2 and S2* α-syn through REST2. Figure 3a,b depicts the distribution of the sum of the hot–hot (HH) and hot–cold (HC) potential energy for S2 and S2*. The observed potential energy overlap translated into an acceptance ratio across replicas of ∼15–20%. Figure 3c,d shows the variation, across replicas, of the HC potential energy (*E*_HC_) with the HH potential energy (*E*_HH_). A significant increase of both *E*_HC_ and *E*_HH_ can be seen for S2 and S2* with an increase of the effective temperature across replicas, as expected. Broadly speaking, the *E*_HC_ varies between −4500 and −2250 kJ mol^–1^ (|Δ*E*_HC_| = 2250 kJ mol^–1^), for S2, and between −5750 and −3500 kJ mol^–1^ (|Δ*E*_HC_| = 2250 kJ mol^–1^), for S2*. Thus, the protein–water potential energy enhancement in S2* results in nearly no overlap between the *E*_HC_ energy in S2 and S2*. The *E*_HH_ energy, in turn, remains nearly unchanged between S2 and S2*. However, the reason is not the fact that protein–protein interactions are not modified in S2*, relative to S2, but rather, the much larger contribution of the ions to the *E*_HH_ energy. Thus, even though protein–protein interactions are not modified in S2*, more extended conformations (expected to occur in S2*) should result in higher potential energies (less negative). This was confirmed by plotting the protein–protein potential energy, *E*_pp_, in Figure 3e,f, where a clear shift to higher energies can be seen. Notice also that *E*_pp_ varies significantly less within each replica for S2*. This indicates that S2* promotes a lower number of protein–protein contacts and therefore also lower protein–protein interaction fluctuations, reducing the sampled potential energy window.

**Figure 4:**
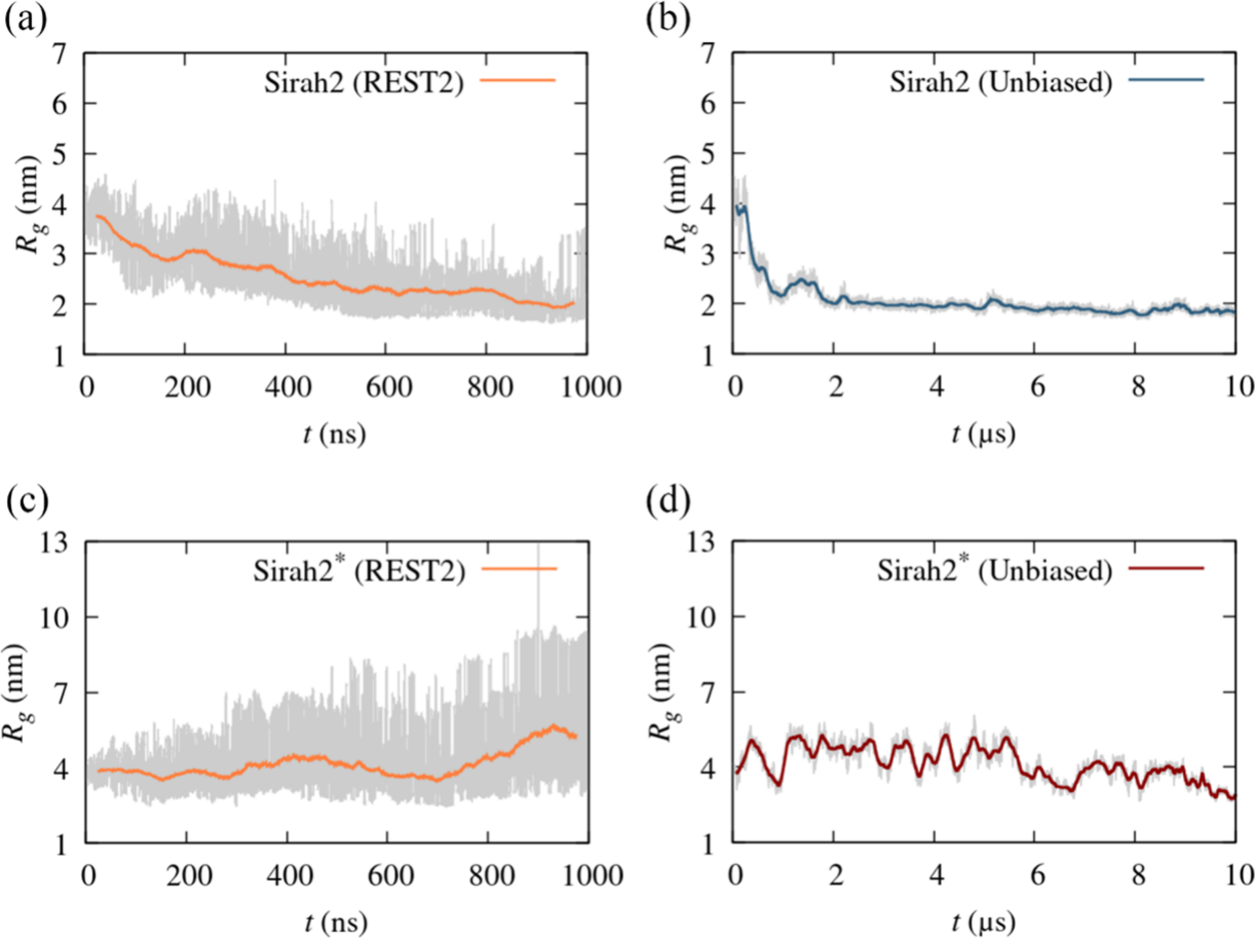
Radius of gyration (*R*_g_) of α-syn along time (a) using REST2 (1 μs) for Sirah2; (b) a 10 μs unbiased MD simulation for Sirah2; (c) using REST2 (1 μs) for Sirah2*; and (d) a 10 μs unbiased MD simulation for Sirah2*.

Figure 4a,b displays the *R*_g_ obtained with REST2 using 30 replicas and 1 μs long trajectories and that obtained through an ordinary (10 μs long trajectory) MD simulation, for S2; Figure 4c,d shows the same results for S2*. As seen in Figure 4a,b, similar results are obtained for the *R*_g_ of S2, although larger fluctuations are observed with REST2, as expected. Although REST2 allows sampling more extended conformations through exchanges with higher effective temperature replicas, a systematic decrease of *R*_g_ is still observed, indicating that extended conformations are not predominant, even at high effective temperatures. This suggests that protein–protein interactions are too strong, or protein–water interactions are too weak. For S2* (Figure 4c,d), significantly more extended conformations are found in both trajectories. Thus, conformations within a similar *R*_g_ range can be observed, suggesting that REST2 may not be necessary to sample the full conformational space of S2* α-syn. Although a lower average *R*_g_ is found from ordinary MD simulation, we found, however, that in REST2, the box for S2* was not large enough to account for some conformations where the protein was completely extended at the highest effective “temperatures;” these conformations appeared roughly after 500 ns, and system size effects are responsible, for instance, the large *R*_g_ peak observed at ∼900 ns.

The increase of protein–water interactions by 30% results in more extended conformations across the replicas. Thus, for some replicas, spurious interactions between the protein and its PBC images influenced the protein’s conformation. A larger box was used to perform the ordinary MD simulation in Figure 4d, where the same problem was initially observed but not in REST2. Nevertheless, our results show that the enhancement of protein–water interactions is needed to observe *R*_g_ values consistent with the experimental values, and therefore, the limitations of S2 regarding the description of IDPs are not statistical. Additionally, the conformational space can be relatively well sampled through ordinary (nonbiased) MD simulation, as expected for a CG FF.

### 3.3 Principal Component Analysis

**Figure 5:**
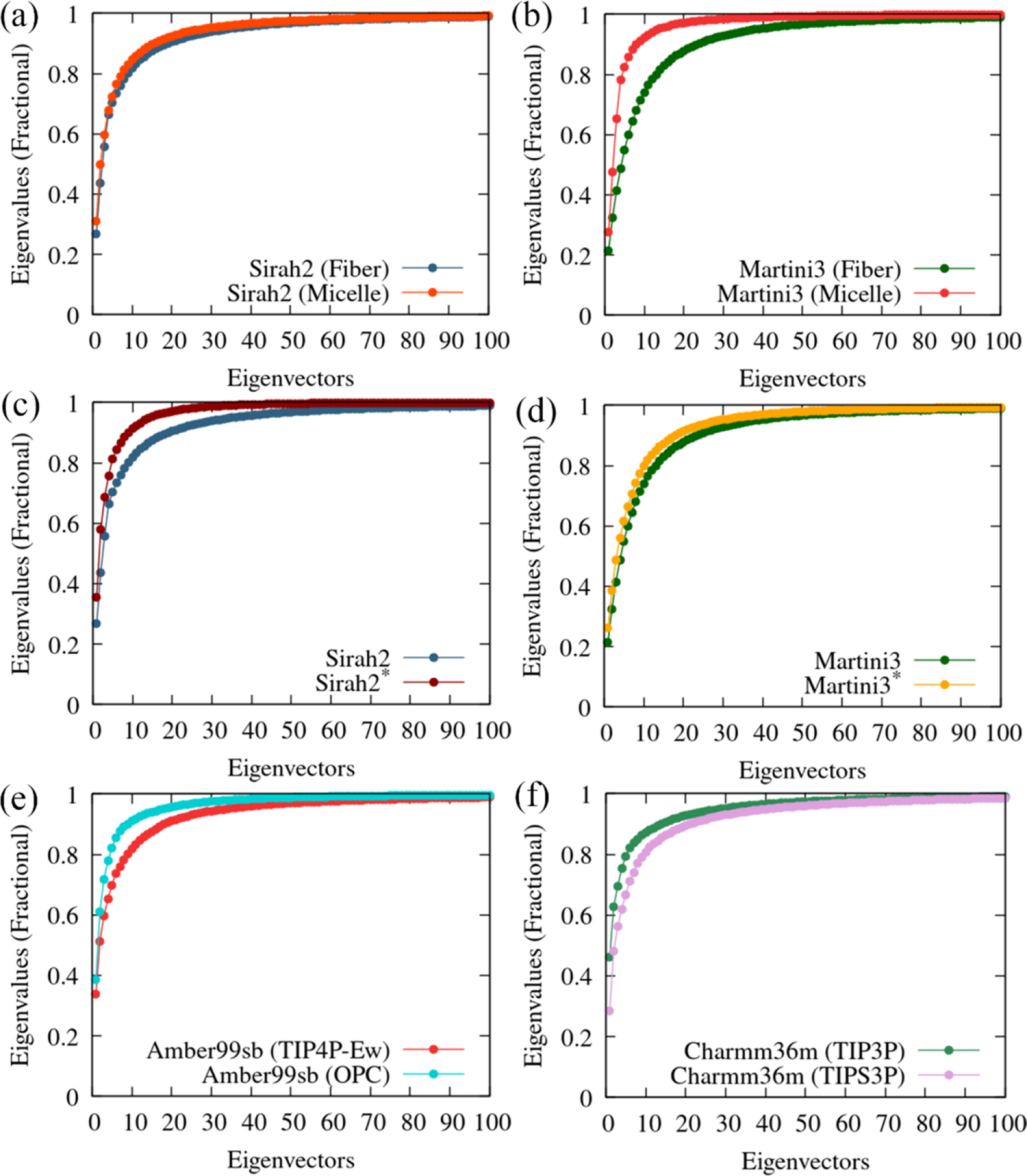
Cumulative sum of the (normalized) eigenvalues for the Cartesian coordinates covariance matrix of α-syn up to the first 100 eigenvalues for (a) Sirah2 and (b) Martini3 starting from a monomer in the fibril (2n0a) and a monomer bound to a micelle (2kkw); (c) for Sirah2 and Sirah2* starting from a fibril; (d) for Martini3 and Martini3* starting from a fibril; (e) for Amber99sb with TIP4P-Ew and OPC; and (f) for Charmm36m with TIP3P and modified TIPS3P. The covariance matrix was computed using the last 5 μs from the CG trajectories; the last 200 ns were used for the amber99sb, and the last 100 ns were used for the charmm36m concerning the all-atom trajectories.

Unlike globular proteins, IDPs are expected to have a much broader essential dynamics subspace because of the heterogeneous conformational space of these proteins. Figure 5a shows that the first two eigenvalues capture only ∼40–50% of the essential subspace for S2. For M3 (Figure 5b), an even lower (∼30%) value is found for the simulation starting from the fiber conformation, and the rate of increase of the eigenvalues’ cumulative sum is lower than that for the micelle starting conformation. Figure 5c,d shows that the scaling versions, S2* and M3* (although to a lesser extent), display a reduction of the number of essential principal components and, therefore, a potential increase of the atomic displacements spanned by the first two eigenvectors. For the all-atom FFs, the Amber99bs/OPC is more similar to the Charmm36m/TIP3P combination, and the sum of the first two eigenvalues is slightly above 60%.

**Figure 6:**
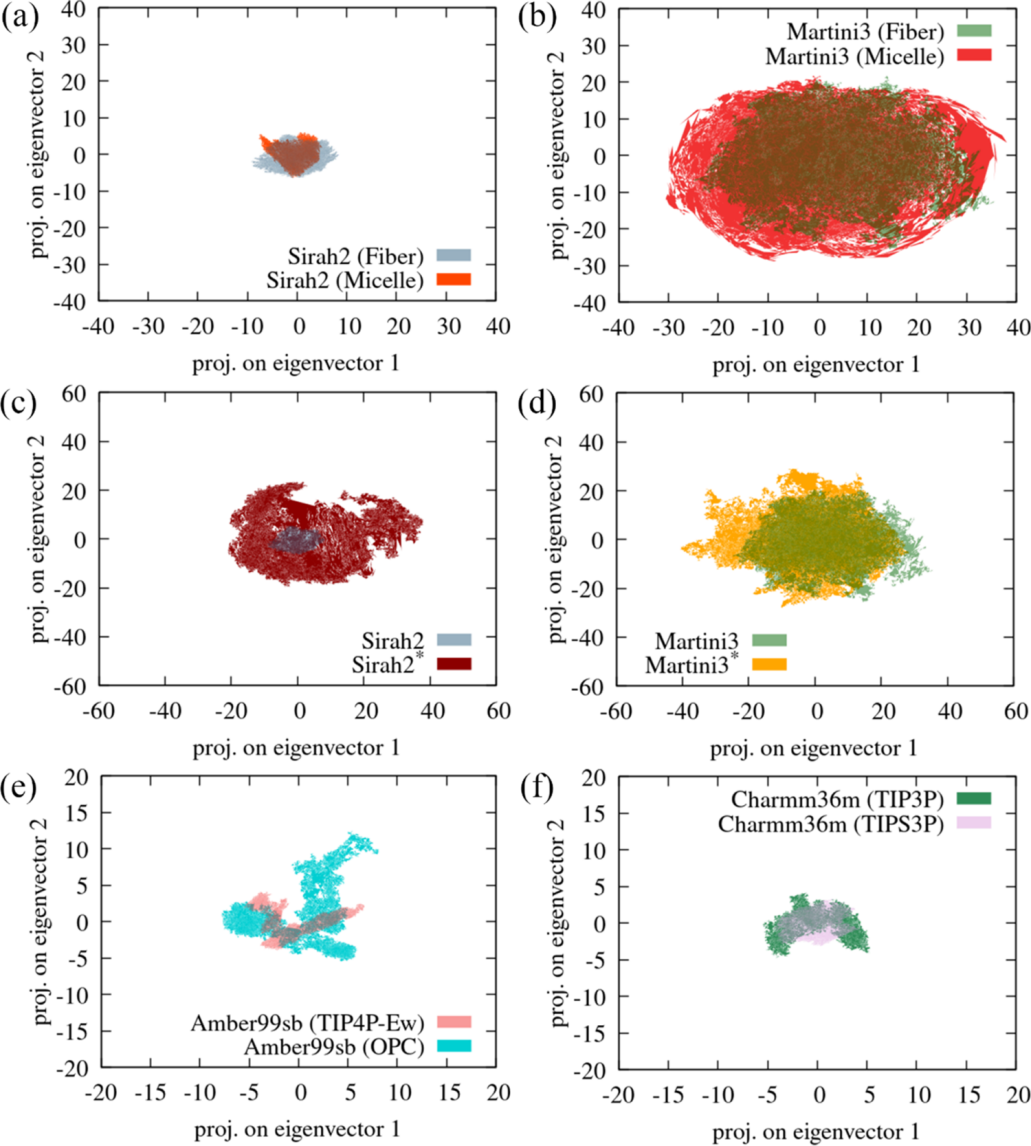
PCA atomic displacement projections of α-syn in the plane formed by the first two eigenvectors for (a) Sirah2 and (b) Martini3 starting from a monomer in the fibril (2n0a) and a monomer bound to a micelle (2kkw); (c) for Sirah2 and Sirah2* (scaled) starting from a fibril; (d) for Martini3 and Martini3* (scaled) starting from a fibril; (e) for Amber99sb with TIP4P-Ew and OPC; and (f) for Charmm36m with TIP3P and modified TIPS3P. Please notice the different scales across the plots.

Figure 6 shows major differences between S2 and M3, concerning the atomic displacement projection within the plane defined by the first two eigenvectors. However, the scaled versions of S2* and M3* depict much more similar projections. This is because the protein–water scaling of M3 is lower (i.e., 10%), resulting in only a slight increase of the protein atomic displacements in the first two eigenvectors plane, whereas that of S2 is larger (i.e., 30%), resulting in a significant increase of the atomic displacement projection.

The atomic displacement projection for the all-atom FFs is more similar to that for S2, as expected from *R*_g_, previously discussed.

These results provide therefore a qualitative picture of the protein mobility, consistent with the magnitude of the fluctuations observed for the *R*_g_. Thus, M3 exhibits a more pronounced mobility than S2, well correlated with the *R*_g_ fluctuations, whereas S2* and M3* exhibit a more similar atomic displacement pattern, which is also consistent with the *R*_g_ fluctuations. The all-atom FFs, in turn, exhibit, atomic displacement projections more consistent with a globular protein.

### 3.4 Fibril Stability

**Figure 7:**
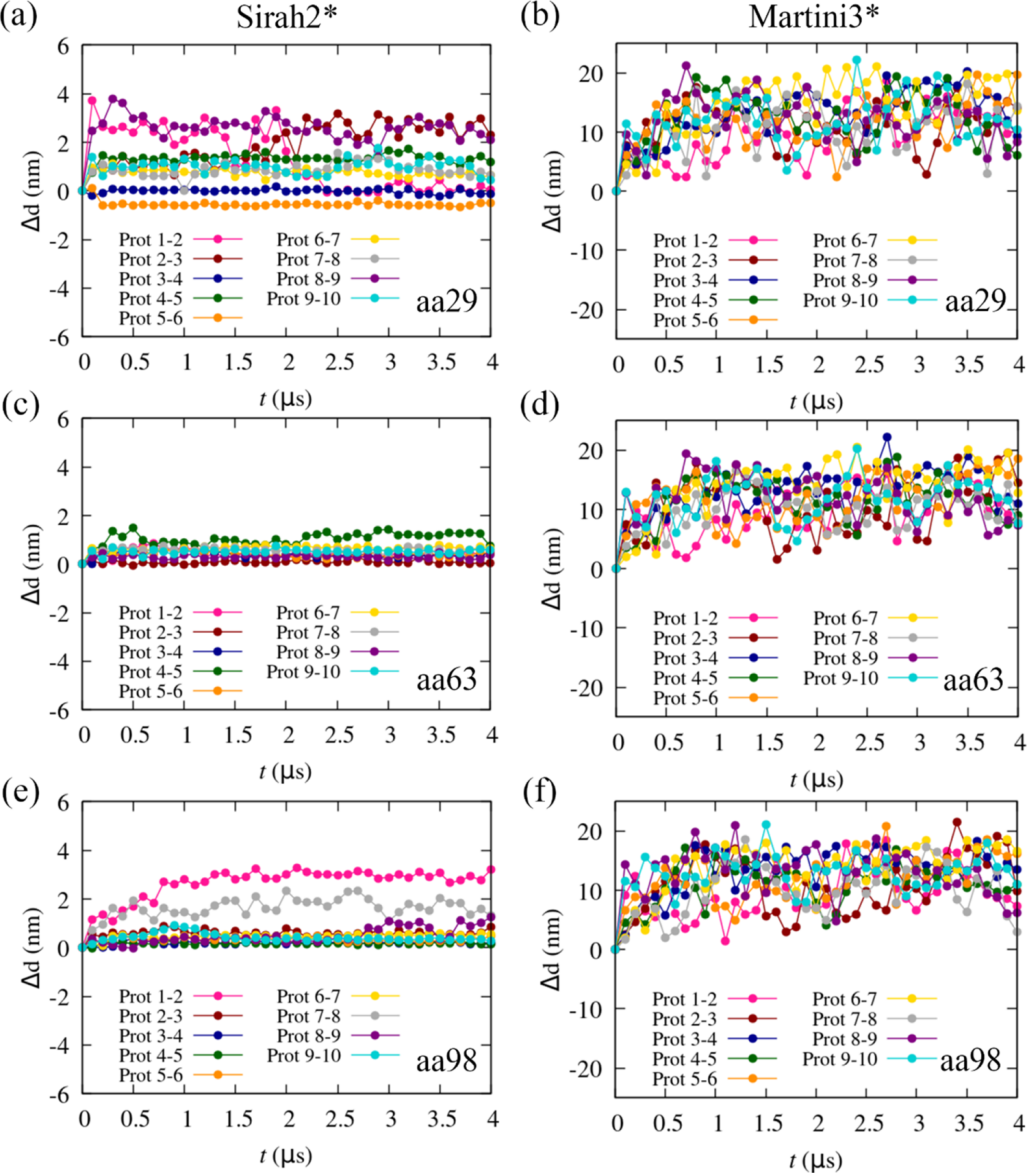
Distance, Δ*d* = *d* – *d*_c_, where *d*_c_ is the distance in the crystal (2n0a),^75^ between specific amino acids in adjacent α-syn monomers in the fibril. The distances were calculated between amino acid 29 for (a) Sirah2* and (b) Martini3*; amino acid 63 for (c) Sirah2* and (d) Martini3*; and amino acid 98 for (e) Sirah2* and (f) Martini3*. Please note the larger scale in Martini3* plots (b), (d), and (f).

We now discuss the stability of an α-syn fibril composed of 10 proteins (2n0a)^75^ with S2* and M3*. These simulations showed that the S2* fibril was stable within a time window of 4 μs, whereas the M3* fibril rapidly disaggregated. We assessed the distance between three amino acids, namely, Ala29, Val63, and Asp 98, which belong to the N-terminal, NAC, and C-terminal regions, respectively. The distances along time, subtracted by the respective distance in the crystal, between these amino acids across contiguous monomers in the fibril are shown in Figure 7. Whereas some modest fluctuations are observed for S2*, and only in the extremities of the fibril (N-terminal and C-terminal), for M3*, the fibril breaks apart with distances between contiguous monomers even in the NAC region, reaching rapidly to 10–20 nm.

Although α-syn does not aggregate spontaneously in water, in vitro, once oligomers form, these are rather stable, and no disaggregation should be expected. Thus, in this sense, the M3* model, although providing a good representation of the monomer, cannot describe oligomers and fibers. S2*, in turn, predicts a stable structure within the MD simulation time frame.

The possible reasons behind this behavior of Martini3 are discussed in the next section.

### 3.5 Potentials of Mean Force

**Figure 8:**
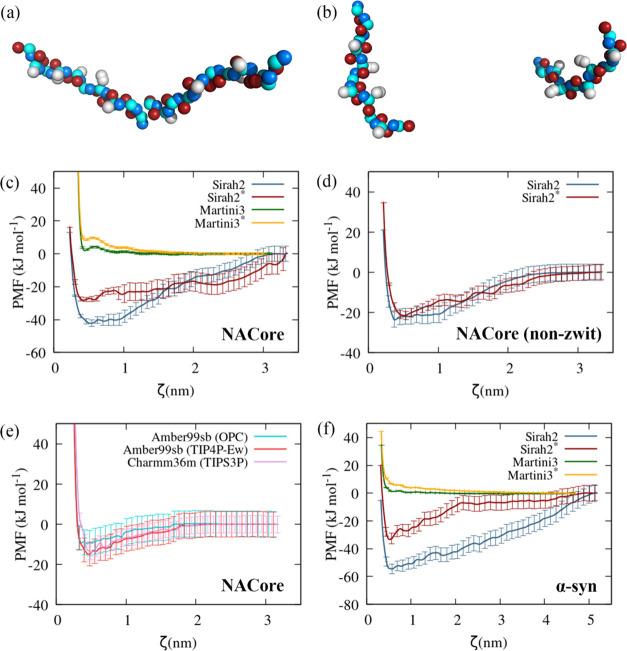
Illustration of the typical orientation of the NACore dimer in the umbrella sampling windows at 2.95 nm with (a) zwitterionic S2* and (b) non-zwitterionic S2*; potentials of mean force (PMF) for (c) zwitterionic NACore with S2, S2*, M3, and M3*; (d) non-zwitterionic NACore with S2 and S2*; (e) zwitterionic NACore with Amber99sb and Charmm36m, and (f) zwitterionic α-syn with S2, S2*, M3, and M3*.

To assess the dimerization binding free energy estimated by the different FFs, we computed the potentials of mean force (PMF) with the CG and all-atom FFs for an 11-mer peptide, namely, NACore^69^ (_68_GAVVTGVTAVA_78_), from the NAC domain of α-syn. The aggregation of this and another similar-sized peptide from the NAC domain were recently studied at room temperature with Amber99sb/TIP4P-Ew.^34,102^ A binding free energy around −15 kJ mol^–1^ was found in those studies. Figure 8a,b displays the orientation of the two NACore peptides at ∼3 nm, modeled with S2*, in the zwitterionic and non-zwitterionic. Figure 8c shows that M3 and M3* do not exhibit any aggregation tendency, whereas S2 and S2* exhibit a long-distance tail. The latter was found to be associated with the orientation of the opposite charge termini of the peptides in the zwitterionic form (Figure 8a). This was confirmed by neutralizing each of the terminal beads by using the same charges of the respective amino acids in nonterminal positions. The respective PMFs (Figure 8d) are in much closer agreement with those observed through all-atom MD simulations (Figure 8e). Somewhat surprisingly, the enhancement of protein–water interactions in S2* does not result in a significant weakening of the binding free energy; this is possibly related to the relatively small size of the peptides. Among the all-atom FFs, the lowest aggregation propensity is observed for Amber99sb/OPC, whereas the PMFs for Amber99sb/TIP4P-Ew and Charmm36m/TIP3SP are similar. Nonetheless, the estimated errors do not allow distinguishing between the respective binding free energies.

The PMFs for α-syn show a similar behavior for M3 and M3*, namely, a nonaggregation tendency, consistent with the disaggregation of the fibril, previously discussed, whereas for S2 and S2*, the binding free energy is, respectively, around ∼−60 and −30 kJ mol^–1^, although for S2, a clear long-distance plateau is not observed up to 55 Å. Thus, for α-syn, clear differences are observed between the binding free energies estimated through S2 and S2*, which are not observed for NACore. The absence of experimental PMFs, however, precludes any conclusions concerning the expected binding free energy of α-syn. Furthermore, we stress that the PMFs of NACore and α-syn are biased to specific dimer conformations, where the distance of the central amino acid of the monomers are restrained, since this was chosen as the reaction coordinate; distinct reaction coordinates would result in different PMFs. Thus, the PMFs only provide information on dimer conformations along this reaction coordinate, ξ, whereas interactions among other amino acids are not restrained. This means that even at long values of ξ, close contacts between the amino acids of the monomers are possible. Our results permit, nevertheless, to unequivocally show that Martini3 cannot describe protein or peptide aggregation, whereas Sirah2 depicts free energy profiles closer to those expected from all-atom FFs.

Concerning the nonaggregation propensity observed for the NACore and α-syn Martini3 models, a recent work by Sasselli and Coluzza^103^ reached similar conclusions for the self-assembly of dipeptides and tripeptides. This was shown to be related to a decrease of the hydrophobicity of the force field, compared with previous parametrizations (Martini2.1 and Martini2.2). Specifically, a decrease in the aggregation propensity of a model diphenylalanine peptide was observed in passing from Martini2.1. to Martini2.2. and from this to Martini3, following an increase of some bead–water interactions compared to bead–bead interactions (see ref (103) for bead details). Furthermore, following Sasselli and Coluzza, bead-type screening allowed showing that “overestimated hydrophilicity arising from charged termini and disruptions in π-stacking interactions due to insufficient planarity in aromatic groups, and a discrepancy in intermolecular distances between this and backbone–backbone interactions,” compromised short peptide self-assembly in Martini3. Whereas some of these effects could be expected^103^ to decrease for large peptides and proteins, our results indicate that Martini3 is also unable to model larger peptides and IDPs’ aggregation. Additionally, whether the reparameterization proposed by Sasselli and Coluzza can solve the problem for proteins while preserving Martini3 protein modeling enhancements deserves further investigation.

**Figure 9:**
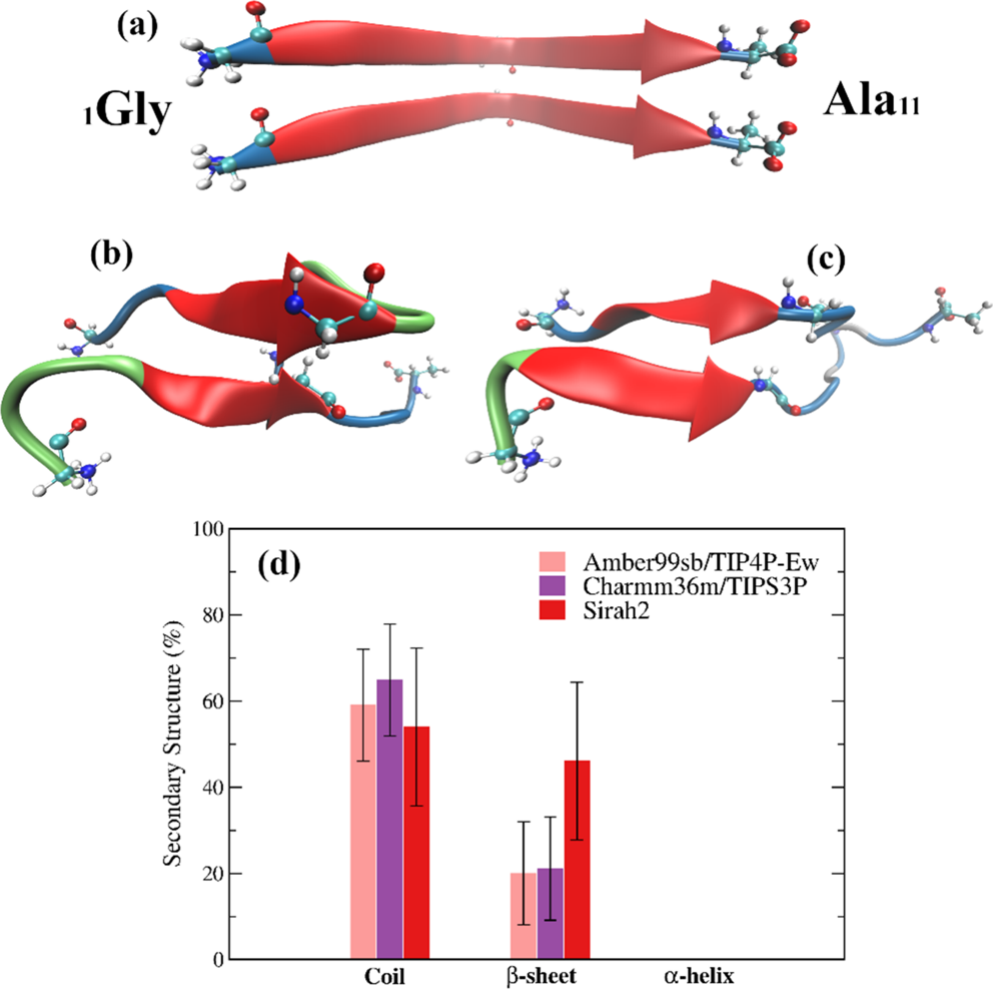
(a) Cartoon representation of the NACore dimer extracted from the crystal structure (2n0a); (b, c) cartoon representation of an umbrella sampling configuration at ξ = 0.55 nm, for Amber99sb/TIP4P-Ew and Charmm36m/TIPS3P, showing the central amino acid (Gly5) used to define ξ; color code: random coil (blue), β-sheet (red), turn (green); and (d) average percentage of random coil, β-sheet, and α-helix (0%) found for Amber99sb/TIP4P-Ew, Charmm36m/TIPS3P, and S2* (non-zwitterionic) along the umbrella sampling trajectory of the window at ξ = 0.55 nm.

We also analyzed the secondary structure of NACore modeled with Amber99sb/TIP4P-Ew, Charmm36m/TIPS3P, and S2* (non-zwitterionic) at different umbrella windows around the minimum in the respective PMF. The initial configuration of the peptides, extracted from the experimental fibril (2n0a), has 70% β-sheet and 26% random coil (see Figure 9a), whereas the same configuration in the Sirah representation has 86% β-sheet and 14% random coil. The secondary structure of the all-atom models was assessed with the program DSSP,^104^ whereas that of S2* was calculated using Sirah’s plugin^105^ for the VMD program.^106^ An energetic criterion (*E* < −0.5 kcal mol^–1^) is used to define backbone NH···O hydrogen-bonds in DSSP. Sirah’s secondary structure is defined as a function of the dihedral angles along the backbone beads, and the residues conformation is assigned into helical, extended, and coil (see ref (63) for the dihedral intervals used to define the types of structure). We compare the extended structure in S2* with the β-sheet estimated by DSSP for the all-atom models.

A maximum of the β-sheet was found at ξ = 0.55 nm for all models, whereas no α-helix was observed for any model. The average percentage of β-sheet found with Sirah2 was 46%, and those for Amber99sb and Charmm36m were, respectively, 20 and 21% (Figure 9b–d). Although the “β-sheet” in S2* is larger by a factor of more than two relative to the all-atom models, the difference between the percentage of β-sheet conserved in the all-atom models and Sirah2* is ∼10%. Thus, S2* NACore loses ∼40% of the β-sheet relative to the same sequence in the fiber, whereas the all-atom models lose ∼50% of the β-sheet. These results show that Sirah’s dimerization binding energy of NACore and the associated structure are consistent with those observed through all-atom MD simulations.

## 4 Conclusions

The use of CG models can be pivotal to the study of complex systems, including disordered proteins’ conformations, protein aggregation pathways, or protein–drug binding. This is especially relevant for large IDPs such as α-syn, which can adopt extended conformations imposing the use of large MD simulation boxes with hundreds of thousands of water molecules. Whereas several all-atom and CG FFs have been modified to reproduce various experimental properties of the monomers, such as the protein radius of gyration, NMR chemical shifts, and J-couplings, less is known about the ability of these models to describe aggregation. A simple approach used to modify CG models to describe IDPs is to increment protein–water interactions, thus favoring less compact conformations. Here, we analyzed the aggregation propensity of two CG FFs and compared it with an all-atom FF developed to model IDPs (Charmm36m) and an unmodified potential, Amber99sb, used to model globular proteins. Our results indicate that the enhancement of protein–water interactions by 30% in S2* provides an enhanced description of the monomer while still providing a description of the aggregation tendency similar to that observed with the all-atom FFs relative to the original S2 model. Martin3, in opposition, although depicting a better agreement with the experimental radius of gyration, exhibits a nonaggregation tendency, further enhanced by the scaling of protein–water interactions by 10%. These results reflect the difficulties in concomitantly describing the conformations of an IDP and its aggregation pathways through all-atom and CG FFs.

Nonetheless, overall, S2* was found to provide a reasonable agreement for the *R*_g_ of α-syn and the aggregation propensity of a small peptide such as NACore, estimated with all-atom FFs, provided the terminal beads are not charged. These results indicate that this force field should be suitable to explore α-syn aggregation pathways and screen protein–drug binding free energies and residence times toward the discovery of aggregation inhibition drug leads for Parkinson’s disease.

## Data Availability

The 3D structures of α-synuclein were downloaded from the Protein Data Bank (PDB IDs: 2n0a and 2kkw). Visualization was performed using VMD (https://www.ks.uiuc.edu/Research/vmd/) and PyMOL (https://www.pymol.org/). GROMACS 2022.3 (https://manual.gromacs.org/) was used for running the MD simulations. The REST2 simulations were performed using GROMACS 2022.3 patched with PLUMED 2.8.1 (https://www.plumed.org/). The REST2 effective temperatures are provided in the Supporting Information (SI). The AMBER99sb and CHARMM36m can be downloaded from https://github.com/intbio/gromacs_ff?tab=readme-ov-file. The sirah2 and martini3 force fields were freely obtained from (https://github.com/SIRAHFF/documentation/releases/tag/GROMACS) and (http://cgmartini.nl/index.php/downloads), respectively. The TIP3SP water model used with Charmm36m was invoked by adding the following line to the mdp simulation file: define = -DUSE_MODIFIED_TIP3P_EPS. The topology files for the scaled versions of sirah2 and martini3, the OPC topology file, and the topologies used for the REST2MD simulations have been deposited in https://github.com/ngalamba/alpha_synuclein/tree/main.

## Supporting Information Available

Molecular simulation studies of distinct α-syn models (Table S1); effective temperatures for the 30 replicas used in REST2 for S2 and S2* (Table S2); radius of gyration (*R*_g_) of α-syn as a function of time for Martini3 (three replicates), starting from a monomer in the fibril (2n0a) and a monomer bound to a micelle (2kkw) (Figure S1); moving average of the RMSD of α-syn along time for the distinct force fields (Figure S2); moving average (MA) of the RMSF of α-syn over time for the distinct force fields (Figure S3) (PDF)
