# Supplementary material for: Wild-Type α-Synuclein Structure and Aggregation: A Comprehensive Coarse-Grained and All-Atom Molecular Dynamics Study
Source: J Chem Inf Model. 2024 Jul 24;64(15):6115–31. doi: 10.1021/acs.jcim.4c00965 (PMC11323248; doi:10.1021/acs.jcim.4c00965)
Supplement: Supplementary file 1 — ci4c00965_si_001.pdf [file ci4c00965_si_001.pdf]

Wild Type α-Synuclein Structure and Aggregation: A Comprehensive Coarse-Grained and All-Atom Molecular Dynamics Study

Supporting Information

Gabriel F. Martins[a], Nuno Galamba[a,*]

[a] BioISI - Biosystems and Integrative Sciences Institute, Faculty of Sciences of the University of Lisbon, C8, Campo Grande, 1749-016 Lisbon, Portugal.

[*]Corresponding author: njgalamba@fc.ul.pt

Table S1 - Molecular simulation studies of distinct α-syn models.

Authors	Year	Model/Simulation Method	Force Field	System
Wu et al.[1]	2009	All-atom/REMD	OPLS-aa/AGBNP	Full-length α-syn
Mirau et al.[2]	2015	CG/Monte Carlo	CG knowledge based interactions	Full-length α-syn
Sahu et al.[3]	2015	All-atom/MD; MM-PBSA	Amberff10/TIP3P	Full-length α-syn
Yu et al.[4]	2015	CG/MD	PACE	Full-length α-syn
Ilie et al.[5] [(a)]	2016	CG/Brownian Dynamics	CG polymorph patchy particle	Sequence of 60 residues of α-syn (30-90)
Mane et al.[6]	2016	All-atom/MD	OPLS-aa/SETTLE	Full-length α-syn
Robustelli et al.[7]	2018	All-atom/MD	a99SB*-ILDN/TIP3P; a03ws/TIP4P-D; a99SB-ILDN/TIP4P-D; a99SB/TIP4P-Ew; a99SB-UCB; Charmm22*/TIP3P; Charmm36m/TIP3P	Full-length α-syn
Baul et al.[8]	2019	CG	SOP-IDP	Full-length α-syn
Balupuri et al.[9]	2019	All-atom/MD	Charmm27/TIP3P	Full-length α-syn
Poma et al.[10]	2019	CG/MD	Coarse-grained developed model in agreement with Gō-like models	Full-length α-syn
Ramis et al.[11]	2019	CG/MD/REST2	Sirah1.0	Full-length α-syn
Mandaci et al.[12]	2020	All-atom/REMD	Amber99SB/ Onufriev–Bashford–Case Generalized Born implicit solvent model	Full-length α-syn
Amos et al.[13]	2021	CG/all-atom/MD	Martini2.1; Charmm36	Full-length α-syn
Kamelabad et al.[14] [(a)]	2021	All-atom/MD	Charmm36/TIP3p; Red and SwissParam	NAC domain of α-syn
Emil Thomasen et al.[15]	2022	CG/MD	Martini3	Full-length α-syn
Pedersen et al.[16]	2022	All-atom/MD	ff19SB/OPC; ff19SB/TIP4P-D; ff03CMAP/TIP4P-D; a99SB-disp/TIP4P-disp	Full-length α-syn
Semenyuk[17]	2022	All-atom/REMD	Charmm36m; CGenFF	Full-length α-syn
Vats et al.[18]	2022	All-atom/MD/docking	OPLS2005/TIP3P	Sequence of 70 residues of α-syn (27-97)
Zhang et al.[19]	2022	All-atom/DMD	Medusa	Full-length α-syn
Boulaamane et al.[20]	2023	All-atom/MD	Charmm36/TIP3P CGenFF	Full-length α-syn
Martins et al.[21] [(a)]	2023	All-atom/MD	Amber99sb/TIP4P-Ew	NACore and NACterm; full-length α-syn
Huang et al.[22] [(a)]	2023	CG/MD	Martini2.2	NACore
Mankoo et al.[23]	2023	All-atom/MD/MM-PBSA	Amber99sb-ILDN/TIP3P	Full-length α-syn
Bruyn et al.[24]	2023	All-atom/MD/REST2	Amber99sb-Disp/TIP4P-D; DES-Amber/TIP4P-D	Full-length α-syn (wt & mutants)
Razzokov et al.[25]	2023	CG/united-atom/MD	Gromos 45a3	Full-length α-syn (mutant fibrils)
Savva et al.[26]	2023	CG/all-atom/MD/accelerated MD	ff03ws/OBC (Born GB modified model)	Full-length α-syn
Smida et al.[27]	2023	All-atom/MD	OPLS-aa-SPC-E	Full-length α-syn
Zhao et al.[28]	2023	All-atom/MD/Steered MD	Amber14sb/TIP3P	Full-length α-syn (fibrils)
Pan et al.[29]	2023	All-atom/Monte Carlo re-weighting	ESFF1 and ff14SB with OPC3-B and other water models	α-syn and other IDPs

[(a)] α-syn-derived models.

Replica Exchange Solute Tempering 2

The scaling factor is represented here by[30] $\lambda = (\beta_m / \beta_0)$ where $\beta_m = 1/k_B T_m$, $\beta_0 = 1/k_B T_0$, T_0 is the temperature of replica 0 (i.e., the temperature of interest), and T_m is the effective temperature of the *m*th replica[30]. Thus, λ is equal to T_0/T_m and changes between 0 and 1. The effective temperatures were obtained through the following exponential law[30],

$$T_m = T_0 e^{m \frac{\ln\left(\frac{T_{n-1}}{T_0}\right)}{n-1}} \tag{S1}$$

where *n* is the number of replicas. This gives a geometric progression[30] of temperatures of ratio 1.013 extending from T_0 =310 K to T_{n-1} = 450 K. **Table S2** shows the values of λ and T_m for the 30 replicas used.

Table S2 - λ values for the 30 replicas used in REST2 for S2 and S2* and the respective effective temperatures.

Replica	λ	Effective *T* (K)	Replica	λ	Effective *T* (K)
0	1	310.00	15	0.825	375.91
1	0.987	314.00	16	0.814	380.77
2	0.974	318.07	17	0.804	385.69
3	0.962	322.19	18	0.793	390.68
4	0.950	326.35	19	0.783	395.73
5	0.938	330.57	20	0.773	400.85
6	0.926	334.85	21	0.763	406.06
7	0.914	339.18	22	0.754	411.29
8	0.902	343.57	23	0.744	416.61
9	0.891	348.01	24	0.735	421.99
10	0.879	352.52	25	0.725	427.45
11	0.868	357.07	26	0.716	432.98
12	0.857	361.69	27	0.707	438.58
13	0.846	366.36	28	0.699	444.25
14	0.835	371.11	29	0.688	450.00

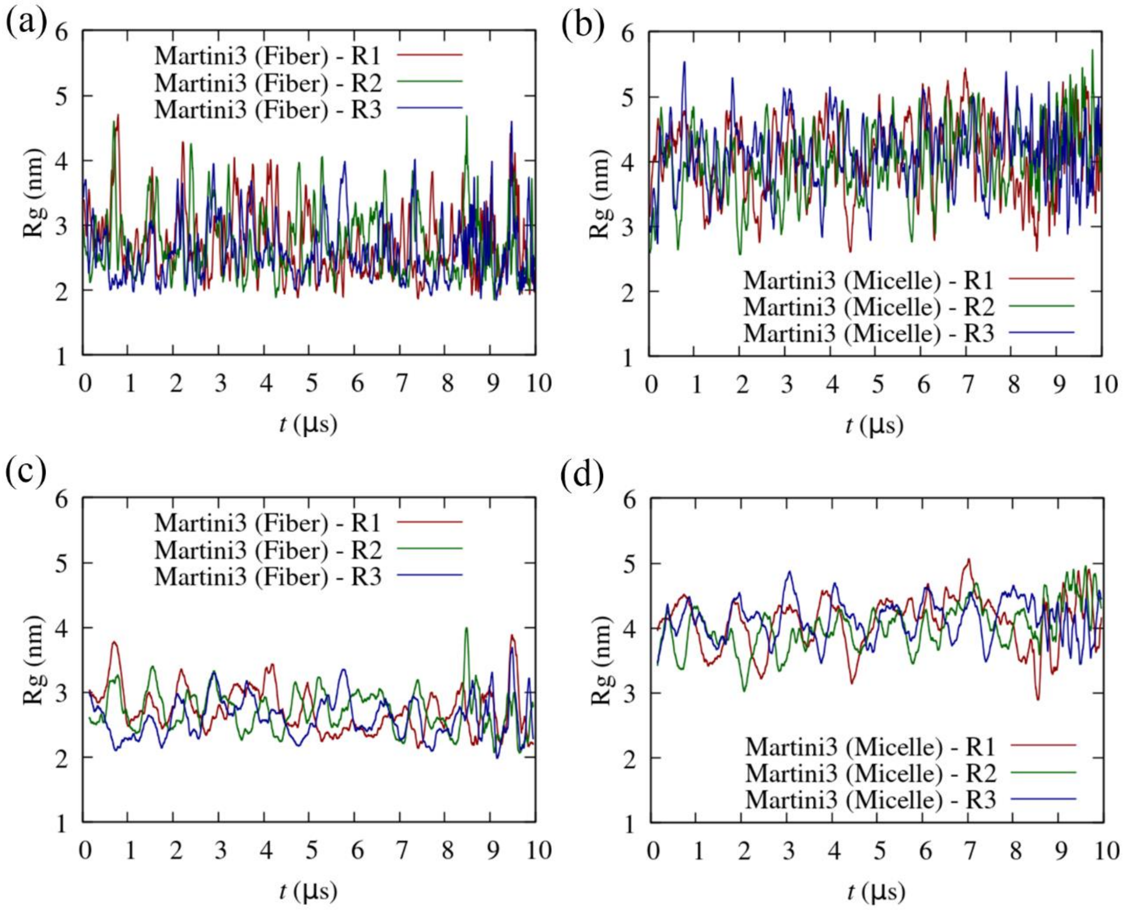

Figure S1 - Radius of gyration (R_g) of α-syn as a function of time for Martini3 (3 replicates), starting from a **(a)** monomer in the fibril (2n0a) and a **(b)** monomer bound to a micelle (2kkw); Moving average of the radius of gyration (R_g) of α-syn **(c)** displayed in **(a)**, and **(d)** displayed in **(b)**.

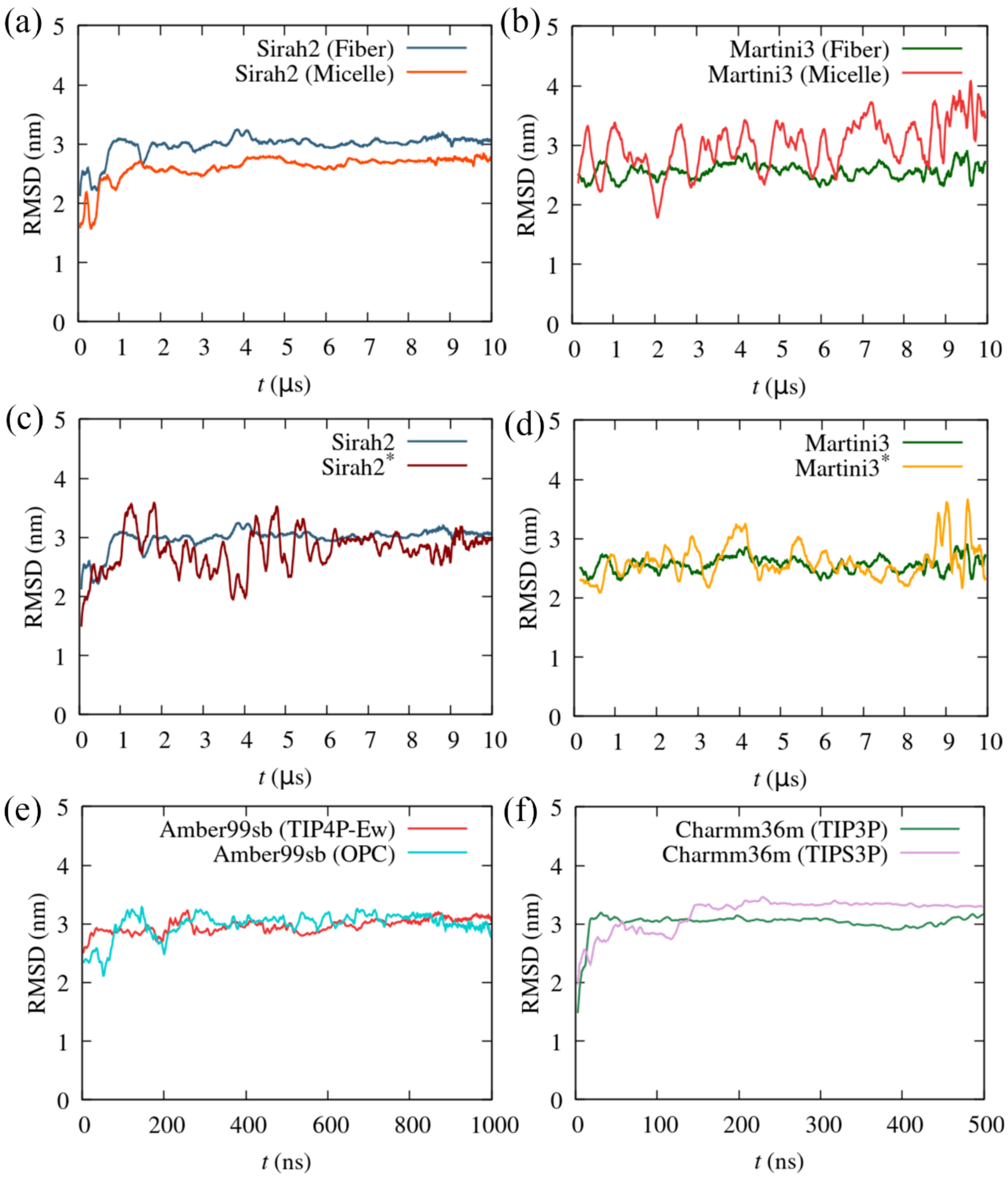

Figure S2 - Moving average (MA) of the RMSD of α-syn along time for **(a)** Sirah2 and **(b)** Martini3, starting from a monomer in the fibril (2n0a) and a monomer bound to a micelle (2kkw); the different order between the RMSD for the fiber and micelle structures at short times in passing from Sirah2 (**a**) to Martini3 **(b)** results only from the MA calculation; **(c)** MA of the RMSD for Sirah2 and Sirah2* starting from a monomer in the fibril; **(d)** MA of the RMSD for Martini3 and Martini3* starting from a monomer in the fibril; **(e)** MA of the RMSD for Amber99sb with TIP4P-Ew and OPC starting from a monomer in the fibril*;* **(f)** MA of the RMSD for C*harmm*36m with TIP3P and modified TIPS3P starting from a monomer in the fibril.

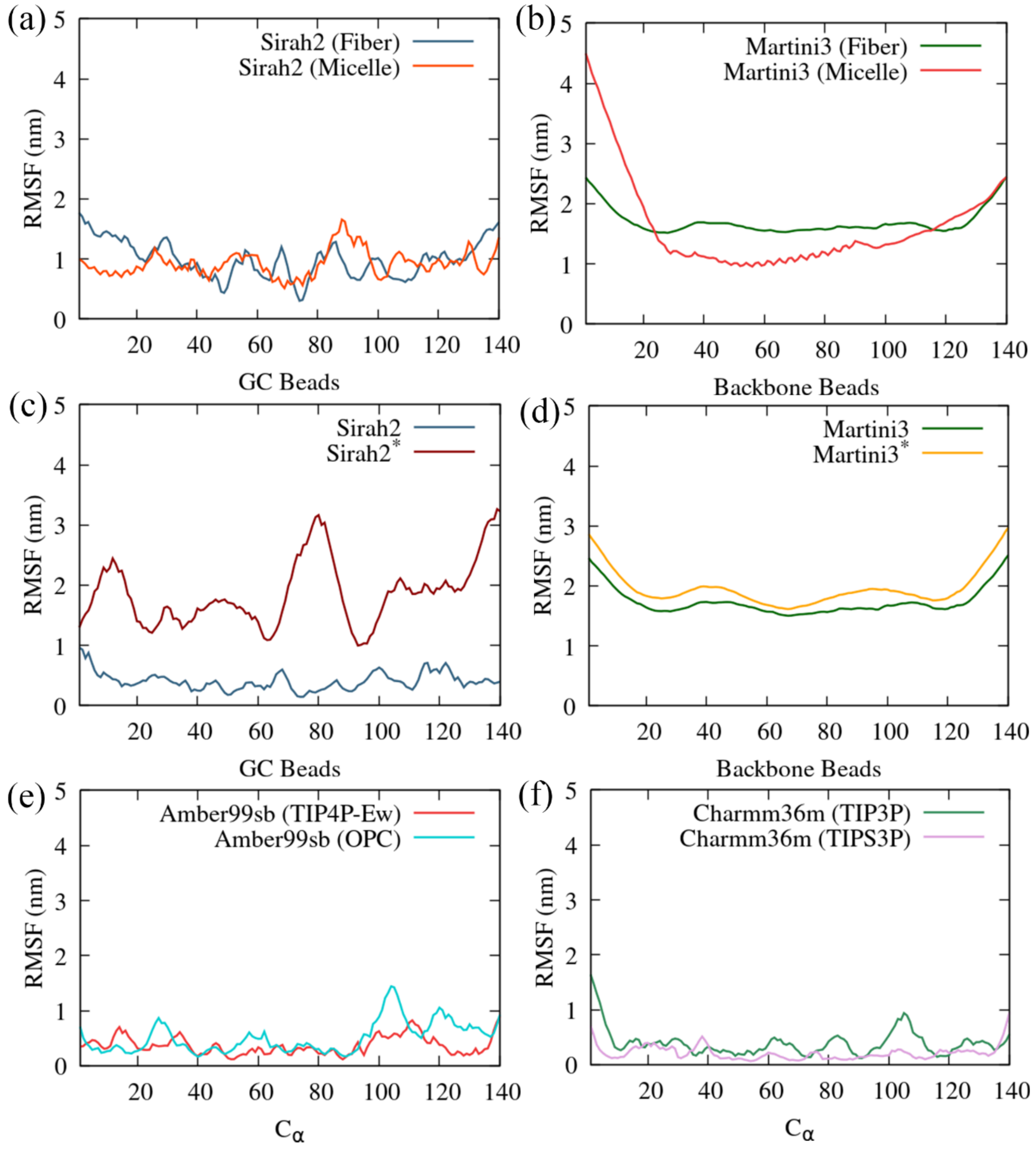

Figure S3 - Moving average (MA) of the RMSF of α-syn along time for **(a)** Sirah2 and **(b)** Martini3, starting from a monomer in the fibril (2n0a) and a monomer bound to a micelle (2kkw); **(c)** MA of the RMSF for Sirah2 and Sirah2* starting from a monomer in the fibril; **(d)** MA of the RMSF for Martini3 and Martini3* starting from a monomer in the fibril; **(e)** MA of the RMSF for Amber99sb with TIP4P-Ew and OPC starting from a monomer in the fibril; **(f)** MA of the RMSF for C*harmm*36m with TIP3P and modified TIPS3P starting from a monomer in the fibril.

References

[1] K.-P. Wu, D.S. Weinstock, C. Narayanan, R.M. Levy, and J. Baum, "Structural reorganization of alpha-synuclein at low pH observed by NMR and REMD simulations," J Mol Biol **391**(4), 784–796 (2009).

[2] P. Mirau, B.L. Farmer, and R.B. Pandey, "Structural variation of alpha-synuclein with temperature by a coarse-grained approach with knowledge-based interactions," AIP Advances **5**(9), 092504 (2015).

[3] K.K. Sahu, M.T. Woodside, and J.A. Tuszynski, "α-Synuclein dimer structures found from computational simulations," Biochimie **116**, 133–140 (2015).

[4] H. Yu, W. Han, W. Ma, and K. Schulten, "Transient β-hairpin formation in α-synuclein monomer revealed by coarse-grained molecular dynamics simulation," The Journal of Chemical Physics **143**(24), (2015).

[5] I.M. Ilie, W.K. den Otter, and W.J. Briels, "A coarse grained protein model with internal degrees of freedom. Application to α-synuclein aggregation," The Journal of Chemical Physics **144**(8), 085103 (2016).

[6] J.Y. Mane, and M. Stepanova, "Understanding the dynamics of monomeric, dimeric, and tetrameric α-synuclein structures in water," FEBS Open Bio **6**(7), 666–686 (2016).

[7] P. Robustelli, S. Piana, and D.E. Shaw, "Developing a molecular dynamics force field for both folded and disordered protein states," Proc Natl Acad Sci U S A **115**(21), E4758–E4766 (2018).

[8] U. Baul, D. Chakraborty, M.L. Mugnai, J.E. Straub, and D. Thirumalai, "Sequence Effects on Size, Shape, and Structural Heterogeneity in Intrinsically Disordered Proteins," The Journal of Physical Chemistry. B **123**(16), 3462 (2019).

[9] A. Balupuri, K.-E. Choi, and N.S. Kang, "Computational insights into the role of α-strand/sheet in aggregation of α-synuclein," Sci Rep **9**(1), 59 (2019).

[10] A.B. Poma, H.V. Guzman, M.S. Li, and P.E. Theodorakis, "Mechanical and thermodynamic properties of Aβ42, Aβ40, and α-synuclein fibrils: a coarse-grained method to complement experimental studies," Beilstein J. Nanotechnol. **10**(1), 500–513 (2019).

[11] R. Ramis, J. Ortega-Castro, R. Casasnovas, L. Mariño, B. Vilanova, M. Adrover, and J. Frau, "A Coarse-Grained Molecular Dynamics Approach to the Study of the Intrinsically Disordered Protein α-Synuclein," J. Chem. Inf. Model. **59**(4), 1458–1471 (2019).

[12] S.Y. Mandaci, M. Caliskan, M.F. Sariaslan, V.N. Uversky, and O. Coskuner-Weber, "Epitope region identification challenges of intrinsically disordered proteins in neurodegenerative diseases: Secondary structure dependence of α-synuclein on simulation techniques and force field parameters," Chemical Biology & Drug Design **96**(1), 659–667 (2020).

[13] S.-B.T.A. Amos, T.C. Schwarz, J. Shi, B.P. Cossins, T.S. Baker, R.J. Taylor, R. Konrat, and M.S.P. Sansom, "Membrane Interactions of α-Synuclein Revealed by Multiscale Molecular Dynamics Simulations, Markov State Models, and NMR," J. Phys. Chem. B **125**(11), 2929–2941 (2021).

[14] M. Rezaei Kamelabad, J. Jahanbin Sardroodi, A. Rastkar Ebrahimzadeh, and M. Ajamgard, "Influence of curcumin and rosmarinic acid on disrupting the general properties of Alpha-Synuclein oligomer: Molecular dynamics simulation," J Mol Graph Model **107**, 107963 (2021).

[15] F.E. Thomasen, F. Pesce, M.A. Roesgaard, G. Tesei, and K. Lindorff-Larsen, "Improving Martini 3 for Disordered and Multidomain Proteins," J. Chem. Theory Comput. **18**(4), 2033–2041 (2022).

[16] K.B. Pedersen, J.C. Flores-Canales, and B. Schiøtt, "Predicting molecular properties of α-synuclein using force fields for intrinsically disordered proteins," Proteins: Structure, Function, and Bioinformatics **91**(1), 47–61 (2023).

[17] P.I. Semenyuk, "REMD Simulations of Full-Length Alpha-Synuclein Together with Ligands Reveal Binding Region and Effect on Amyloid Conversion," Int J Mol Sci **23**(19), 11545 (2022).

[18] S. Vats, R. Kondabala, and S. Saxena, "Identification of alpha-Synuclein Disaggregator from Camellia sp. Insight of Molecular Docking and Molecular Dynamics Simulations," ChemistrySelect **7**(10), e202104131 (2022).

[19] Y. Zhang, Y. Wang, Y. Liu, G. Wei, F. Ding, and Y. Sun, "Molecular Insights into the Misfolding and Dimerization Dynamics of the Full-Length α-Synuclein from Atomistic Discrete Molecular Dynamics Simulations," ACS Chem. Neurosci. **13**(21), 3126–3137 (2022).
[20] Y. Boulaamane, K. Jangid, M.R. Britel, and A. Maurady, "Probing the molecular mechanisms of α-synuclein inhibitors unveils promising natural candidates through machine-learning QSAR, pharmacophore modeling, and molecular dynamics simulations," Mol Divers, (2023).
[21] G.F. Martins, C. Nascimento, and N. Galamba, "Mechanistic Insights into Polyphenols' Aggregation Inhibition of α-Synuclein and Related Peptides," ACS Chem Neurosci **14**(10), 1905–1920 (2023).
[22] R. Huang, R. Tang, X. Song, J. Wang, K. Chen, and W. Tian, "Insights into aggregation dynamics of NACore peptides from coarse-grained simulations," Proteins: Structure, Function, and Bioinformatics **91**(1), 16–21 (2023).
[23] O.K. Mankoo, A. Kaur, D. Goyal, and B. Goyal, "Unravelling the destabilization potential of ellagic acid on α-synuclein fibrils using molecular dynamics simulations," Phys. Chem. Chem. Phys. **25**(11), 8128–8143 (2023).
[24] E. de Bruyn, A.E. Dorn, O. Zimmermann, and G. Rossetti, "SPEADI: Accelerated Analysis of IDP-Ion Interactions from MD-Trajectories," Biology **12**(4), 581 (2023).
[25] J. Razzokov, S. Fazliev, M. Makhkamov, P. Marimuthu, A. Baev, and E. Kurganov, "Effect of Electric Field on α-Synuclein Fibrils: Revealed by Molecular Dynamics Simulations," International Journal of Molecular Sciences **24**(7), 6312 (2023).
[26] L. Savva, and J.A. Platts, "How Cu(II) binding affects structure and dynamics of α-synuclein revealed by molecular dynamics simulations," Journal of Inorganic Biochemistry **239**, 112068 (2023).
[27] K. Smida, M.A. Albedah, R.F. Rashid, and A.-R. Al-Qawasmi, "Molecular dynamics method for targeting α-synuclein aggregation induced Parkinson's disease using boron nitride nanostructures," Engineering Analysis with Boundary Elements **146**, 89–95 (2023).
[28] N. Zhao, Q. Zhang, F. Yu, X. Yao, and H. Liu, "The α-Synuclein Monomer May Have Different Misfolding Mechanisms in the Induction of α-Synuclein Fibrils with Different Polymorphs," Biomolecules **13**(4), 682 (2023).
[29] Z. Pan, J. Mu, and H.-F. Chen, "Balanced Three-Point Water Model OPC3-B for Intrinsically Disordered and Ordered Proteins," J. Chem. Theory Comput. **19**(15), 4837–4850 (2023).
[30] G. Bussi, "Hamiltonian replica exchange in GROMACS: a flexible implementation," Molecular Physics **112**(3–4), 379–384 (2014).
